# Ionomycin Exhibits Potent and Selective Bactericidal Activity Against *Clostridioides difficile* Through Calcium‐Dependent Membrane Disruption

**DOI:** 10.1002/mbo3.70269

**Published:** 2026-03-17

**Authors:** Ahmed A. Abouelkhair, Nader S. Abutaleb, Mohamed N. Seleem

**Affiliations:** ^1^ Department of Biomedical Sciences and Pathobiology, Virginia‐Maryland College of Veterinary Medicine Virginia Polytechnic Institute and State University Blacksburg Virginia USA; ^2^ Center for One Health Research, Virginia Polytechnic Institute and State University Blacksburg Virginia USA; ^3^ Department of Microbiology and Immunology Faculty of Pharmacy, Zagazig University Zagazig Al‐Sharqia Egypt

**Keywords:** bacterial membrane polarization, *C. difficile* infection, calcium influx, ionophores, repurposing, toxin inhibition

## Abstract

*Clostridioides difficile* represents a critical global health concern due to its high morbidity, mortality, and recurrent infections associated with current therapeutic options. There is an urgent need for novel, selective anti‐*C. difficile* therapeutic agents. Screening the microbial metabolite library against *C. difficile* identified ionomycin, a calcium ionophore produced by *Streptomyces conglobatus*, as a potent inhibitor for *C. difficile*. Ionomycin exhibited potent activity against 30 *C. difficile* isolates, with minimum inhibitory concentrations of 1 μg/mL and 2 μg/mL against 50% (MIC_50_) and 90% (MIC_90_) of isolates, respectively. Time‐kill assays revealed rapid bactericidal activity, achieving a ≥ 3 log₁₀ reduction within 8 h, surpassing the efficacy of vancomycin and fidaxomicin. At subinhibitory concentrations, ionomycin markedly reduced toxin production (~20%) and spore formation (~3 log_10_ CFU/mL). Moreover, ionomycin exerted a potent activity against *C. difficile* spore germination and significantly prevented the toxin production from the germinating *C. difficile* cells. Importantly, ionomycin displayed limited activity against representative gut microbiota strains, indicating a favorable selectivity profile. Mechanistic investigations revealed a calcium‐dependent mode of action, as exogenous calcium enhanced ionomycin‐mediated bactericidal activity, whereas calcium chelation attenuated its effects. Consistent with this mechanism, ionomycin disrupted *C. difficile* membrane potential, an effect that was further potentiated by calcium supplementation. Collectively, these findings identify ionomycin as a potent and selective anti‐*C. difficile* agent with a distinct calcium‐dependent mechanism of action, supporting its potential as a promising therapeutic candidate warranting further investigation.

## Graphical abstract

Ionomycin exhibits potent and selective bactericidal activity against *Clostridioides difficile*, rapidly reducing vegetative cells, toxin production, sporulation, and spore germination while sparing representative gut microbiota. Its activity is calcium‐dependent and mediated through disruption of bacterial membrane potential, highlighting ionomycin as a promising lead molecule for further development against CDI.

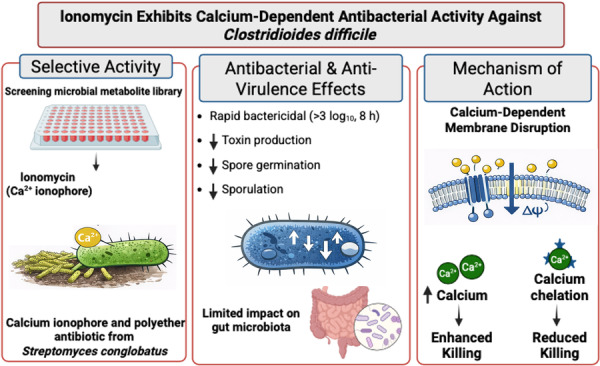

## 1 Introduction

*Clostridioides difficile* is the leading cause of healthcare‐associated infections and considered as an urgent health threat worldwide. Infections caused by *C. difficile* range from mild diarrhea to severe pseudomembranous colitis, toxic megacolon, sepsis, and intestinal barrier dysfunction (Lev 2023; Feuerstadt et al. 2023). According to the Centers for Disease Control and Prevention (CDC), in 2019, *C. difficile* infection (CDI) accounted for approximately 223,900 cases in the United States, resulting in 12,800 deaths and nearly $1 billion in healthcare costs (Feuerstadt et al. 2023; CDC 2022). Furthermore, the CDC's 2024 Emerging Infections Program (EIP) report indicated that there were 116.1 CDI cases per 100,000 individuals across all EIP surveillance sites, including both community‐acquired and healthcare‐acquired settings. Consequently, *C. difficile* has been classified by the CDC as an “urgent threat,” representing the highest level of public health concern, that necessitates development of new therapeutics (Lessa et al. 2015a).

Pathogenesis of CDI is primarily driven by the production of two major toxins, toxin A (TcdA) and toxin B (TcdB), which induce mucosal inflammation, epithelial damage, and profuse diarrhea (Di Bella et al. 2016). Hypervirulent strains, such as NAP1/027, are characterized by overproduction of these toxins and expression of a third binary toxin (CDT), contributing to increased disease severity and transmission (Gerding et al. 2014). NAP1/027 strains were reported to be responsible for approximately 31% of hospital‐acquired and 19% of community‐acquired CDI cases (Lessa et al. 2015b). Another critical virulence factor of *C. difficile* is spore formation. *C. difficile* spores can persist on environmental surfaces for up to 5 months, promoting transmission and infection recurrence (Castro‐Córdova et al. 2021; Smith et al. 2020).

Recurrence rates of CDI are alarmingly high, with 50%–60% of recovered patients experiencing a relapse of CDI within a few weeks of ceasing treatment, leading to a significant increase in hospitalization and treatment cost (Feuerstadt et al. 2023; Barbut et al. 2000; Cole and Stahl 2015). Currently, vancomycin (VAN) and fidaxomicin (FDX) are the only FDA‐approved antibiotics for treatment of *C. difficile* infections. Metronidazole (MTZ), previously used for the treatment of CDIs in adults, is now restricted to non‐severe cases when VAN or FDX are unavailable (McDonald et al. 2018). However, both VAN and MTZ lack sporicidal activity, contributing to recurrence in 25%–30% of treated patients and treatment failure rates up to 14% and 22%, respectively (Vardakas et al. 2012; Cornely et al. 2012; Viswanathan et al. 2010). Although FDX demonstrates greater selectivity toward *C. difficile* and reduces sporulation compared with VAN and MTZ, recurrence rates remain as high as 15.4%, and up to 27.1% in infections caused by hypervirulent ribotype 027 strains (Babakhani et al. 2012a; Louie et al. 2011). These limitations underscore the urgent need for novel, selective therapeutics targeting *C. difficile* and its unique virulence mechanisms (Antrim 2022; Pal et al. 2023; Tsigrelis 2020).

Screening of microbial metabolites represents a powerful and efficient strategy for identifying bioactive compounds with antibacterial potential. Microbial secondary metabolites have historically served as a rich source of antibiotics, often exhibiting unique chemical scaffolds and mechanisms of action that are difficult to access through synthetic approaches (Rudrapal et al. 2020; Sexton 2022; Nosengo 2016; Abutaleb and Seleem 2020a). Through a targeted screen of a microbial metabolite library, we identified ionomycin (Abouelkhair and Seleem 2024), a calcium ionophore and polyether antibiotic derived from *Streptomyces conglobatus*, as a potent inhibitor of *C. difficile*. Although ionomycin has been previously reported to exhibit antibacterial activity against Gram‐positive bacteria, with limited effects on Gram‐negative species (Liu et al. 1978), its activity against *C. difficile* and its impact on disease‐relevant phenotypes have not been comprehensively characterized. In the present study, we evaluated the antibacterial activity of ionomycin against a diverse panel of *C. difficile* clinical isolates, assessed its killing kinetics, and examined its selectivity toward representative members of the human gut microbiota. Furthermore, we investigated ionomycin's effects on key *C. difficile* virulence factors, including toxin production, spore formation, and spore germination, and conducted mechanistic studies to elucidate its calcium‐dependent mode of action and membrane‐depolarizing effects.

## 2 Materials and Methods

### 2.1 Bacterial Strains, Drugs, Media and Chemicals

*C. difficile* and representative gut microbiota isolates used in this study (Tables S1 and S2) were obtained from the Biodefense and Emerging Infections Research Resources Repository (BEI Resources), the American Type Culture Collection (ATCC), and the Centers for Disease Control and Prevention (CDC). Drugs were purchased commercially: ionomycin (Cayman chemicals), FDX (Biosynth Carbosynth), MTZ (Alfa Aesar), and VAN (Gold Biotechnology).

Media and reagents were purchased from commercial vendors: brain heart infusion broth and de Man‐Rogosa‐Sharpe (MRS) broth (Becton Dickinson), yeast extract (Fisher Scientific), l‐cysteine (Alfa Aesar), glycine, vitamin K, calcium chloride and hemin (Sigma‐Aldrich), and taurocholic acid sodium (TA) (Chem Impex), Eagle's Minimum Essential Medium (MEM) (ATCC),3‐(4,5‐dimethylthiazol‐2‐yl)‐5‐(3‐carboxymethoxyphenyl)‐2‐(4‐sulfophenyl)‐2H‐tetrazolium) (MTS) (Promega), bis‐(1,3‐Dibarbituric acid)‐trimethine oxanol (DiBAC4(3)) (Biotium), 3,12‐*bis*(carboxymethyl)‐6,9‐dioxa‐3,12‐diazatetradecanedioic acid (Ethylene Glycol Tetraacetic Acid (EGTA)) (Cayman chemicals), Fura‐2 AM (ThermoFisher), and phosphate‐buffered saline (PBS) (Corning).

### 2.2 Minimum Inhibitory Concentrations (MICs) of Ionomycin Against *C. difficile*

The MICs of ionomycin were determined against a panel of *C. difficile* isolates as described previously (Abouelkhair and Seleem 2024; Naclerio et al. 2020; Seleem et al. 2026; Ammara et al. 2025). Ionomycin and control antibiotics were serially diluted in brain heart infusion broth (BHI) supplemented with yeast extract, resazurin, hemin, vitamin K₁, and l‐cysteine. Assays were performed in sterile 96‐well microtiter plates under anaerobic conditions. A bacterial suspension equivalent to 0.5 McFarland standard was prepared and further diluted to yield an inoculum of approximately 5 × 10⁵ CFU/mL, which was added to each well. Plates were then incubated at 37°C anaerobically (inside the anaerobe container jars containing BD GasPak anaerobic sachets) for 48 h. The MICs were determined as the lowest concentrations which completely inhibited the bacterial growth as determined visually (Abutaleb and Seleem 2020b). The minimum bactericidal concentration (MBC) was determined by plating 10 µL aliquots from wells showing no visible growth onto pre‐reduced BHIS agar plates. Plates were incubated anaerobically at 37°C for 48 h, and the MBC was defined as the lowest concentration producing a ≥ 99.9% reduction in the initial bacterial count (Arthithanyaroj et al. 2021; Kotb et al. 2018).

### 2.3 Time‐Kill Kinetics of Ionomycin

The time kill assay was performed to evaluate how potent anti‐*C. difficile* activity ionomycin exhibits against *C. difficile*, as previously described (Pal and Seleem 2022; Abouelkhair et al. 2025; Holly et al. 2025). Briefly, logarithmic phase cultures of *C. difficile* ATCC 43255 (ribotype 087) and ATCC 630 (ribotype 012) were diluted to approximately 10⁶ CFU/mL in pre‐reduced BHIS broth. Ionomycin (3× MIC) and the control antibiotics (VAN, MTZ, and FDX) (each at 5× MIC) were added to the cultures and incubated anaerobically at 37°C. DMSO‐treated cultures served as negative controls. Aliquots were withdrawn at 0, 4, 8, 12, 24, and 48 h, serially diluted in PBS, and plated on pre‐reduced BHIS agar plates. Following anaerobic incubation at 37°C, the bacterial colony‐forming units (CFU) were calculated.

### 2.4 Cell Cytotoxicity Assay

To evaluate the potential toxic effect of ionomycin, cytotoxicity assay was performed against cell lines as has been described previously (Pal and Seleem 2022; Pal et al. 2021). Kidney (Vero) cell lines (ATCC) and Human epithelial colorectal (Caco‐2) cell lines (ATCC) were cultured at 37°C, 5% CO₂ in EMEM supplemented with 10% FBS (Vero) or 20% FBS (Caco‐2) or and 1% penicillin–streptomycin. Cells were seeded into treated 96‐well plates (80%–90% confluence) and exposed to ionomycin (up to 64 µg/mL). DMSO controls matched vehicle concentration. After 24 h, cell viability was quantified using an MTS/PMS assay with absorbance read at 490 nm. Percent viability was calculated relative to vehicle controls.

### 2.5 Toxin Inhibition Assay

To investigate ionomycin's effect on *C. difficile* toxin production, TcdA and TcdB levels were measured as previously described (Abutaleb and Seleem 2020b; Pal and Seleem 2022, 2020). Log‐phase cultures of *C. difficile* ATCC 43255 were treated with ionomycin, VAN or FDX at subinhibitory concentrations corresponding to 0.125× and 0.25× MIC (in triplicate). Following incubation, culture supernatants were collected, and concentrations of TcdA and TcdB were quantified using a commercial ELISA kit (tgc BIOMICS, Germany) according to the manufacturer's instructions. Absorbance was recorded at 450 nm with background correction at 620 nm using a BioTek Synergy H1 Hybrid microplate reader. Absorbance values (OD₄₅₀–OD₆₂₀) were used to determine relative toxin levels compared with untreated controls.

To confirm that changes in toxin levels were not attributable to differences in bacterial growth, bacterial viability was assessed by CFU enumeration following plating on pre‐reduced BHIS agar.

### 2.6 Spore Inhibition Assay

The effect of ionomycin on *C. difficile* spore formation was assessed following the procedure described earlier (Abutaleb and Seleem 2020b; Pal and Seleem 2023). Log‐phase culture of *C. difficile* ATCC 43255 was diluted in BHIS to an initial density of ~10^6^ CFU/mL. The bacterial solution was then distributed into tubes containing test agents; ionomycin, VAN, MTZ, and FDX, at subinhibitory concentrations of 0.25× and 0.5× MIC (in triplicate) and incubated anaerobically at 37°C for 6 days. To determine the total bacterial counts (vegetative bacteria plus spores), an aliquot from each tube was diluted and plated on pre‐reduced BHIS agar plates supplemented with 0.1% taurocholic acid. Thereafter, the remaining solution of each tube was centrifuged, and the pellets were suspended in PBS and heated at 65°C for 40 min, then refrigerated overnight at 4°C to kill the vegetative cells. The heat‐resistant spore counts were calculated by serially diluting the resultant solutions and plating onto pre‐reduced BHIS agar supplemented with 0.1% taurocholic acid.

### 2.7 Spore Outgrowth Assay Under Germination Permissive Conditions

*C. difficile* ATCC 43255 was cultured anaerobically on pre‐reduced BHIS agar plates for 48 h at 37°C. Colonies were scraped and spread onto pre‐reduced 70:30 agar plates for sporulation (Edwards and McBride 2016). After incubation for 6 days at 37°C under anaerobic conditions, the spores were harvested by suspending them in PBS, followed by two washes with PBS. The suspension was then heat‐treated at 65°C for 45 min to eliminate any remaining vegetative cells.

The spore outgrowth assay was conducted as previously described (Chilton et al. 2016; Allen et al. 2013). Spores of *C. difficile* ATCC 43255 were incubated anaerobically at 37°C in pre‐reduced BHIS broth containing germinants (0.2% TA and 50 mM glycine) in the presence of ionomycin or the control antibiotics (VAN, MTZ, and FDX) at concentrations equivalent to 5× MIC (in triplicate). DMSO, with or without germinant, served as controls. At designated time points (0, 2, 4, 6, 8, 10, 12, and 14 days), 100 µL aliquots were collected, serially diluted, and plated on pre‐reduced BHIS agar supplemented with 0.1% TA to determine total viable counts (vegetative cells + spores). To quantify heat‐resistant spores, additional aliquots were heated at 65°C for 45 min before plating on the same medium.

To verify germination and toxin production, a Vero cell cytotoxicity assay was performed as described previously (Roshan et al. 2018). Briefly, aliquots (10 µL) from each time point (prior to heat treatment) were centrifuged, diluted 10‐fold in MEM, and applied to confluent Vero cell monolayers. The cells were incubated for 24 h at 37°C, followed by examination under a differential interference contrast (DIC) microscope. Cytopathic effects were quantified by assessing the percentage of cell rounding relative to untreated control samples.

### 2.8 Antibacterial Activity of Ionomycin Against Human Intestinal Microflora

The MICs of ionomycin were determined against representative members of the human gut microflora as previously described (Abutaleb and Seleem 2020b; Stolz et al. 2024). A bacterial suspension equivalent to 0.5 McFarland was prepared and diluted in BHIS broth (for *Bifidobacterium* and *Bacteroides*) or MRS broth (for *Lactobacillus*), to achieve a bacterial concentration of ~5 × 10^5^ CFU/mL. Plates containing *Bifidobacterium* and *Bacteroides* were incubated anaerobically at 37°C for 48 h, while plates containing *Lactobacillus* were incubated at 37°C in the presence of 5% CO₂ for 48 h, before determining the MICs.

### 2.9 Calcium Supplementation Assay

Given that ionomycin is a calcium ionophore facilitating transmembrane transport of Ca²⁺, which may contribute to bactericidal activity (Liu and Hermann 1978), the impact of calcium supplementation on ionomycin's anti‐*C. difficile* activity was assessed. Log‐phase culture of *C. difficile* was diluted in pre‐reduced BHIS to ~10^6^ CFU/mL. Ionomycin was added at 3× MIC, and calcium salt (CaCl_2_) was supplemented at final concentrations of (25, 50, 75, and 100 mM). Daptomycin (5× MIC) was included as a positive control, as it has a calcium‐dependent bactericidal mechanism. VAN and FDX (5× MIC each) were used as negative controls, given their calcium‐independent modes of action. A no‐drug treatment served as the growth control. Aliquots were collected at 0‐, 2‐, and 4‐h post‐treatment, serially diluted in PBS, and plated onto pre‐reduced BHIS agar plates. Bacterial CFU were enumerated after 48 h of anaerobic incubation at 37°C to determine bacterial viability under each treatment condition.

### 2.10 Measurement of Intracellular Calcium Levels (Ca^2+^)

The intracellular calcium concentrations were measured using the fluorescent calcium indicator fura‐2 acetoxymethyl ester (fura‐2 AM) (ThermoFisher) as previously reported (Clementi 2014). A log‐phase culture of *C. difficile* ATCC 43255 was centrifuged and washed twice with pre‐reduced BHIS (OD_600_ of ~1). Bacteria were first exposed to 5 µM fura‐2 AM and incubated anaerobically to allow dye loading. Fluorescence was recorded at dual excitation/emission wavelengths (340/510 nm for “Value 1” and 380/510 nm for “Value 2”) using a BioTek Synergy H1 Hybrid plate reader and black 96‐well plates (ThermoFisher, Cat. No. 267342), which are compatible with far‐UV excitation. Subsequently, ionomycin (5× MIC) was added with or without calcium supplementation (100 mM CaCl₂), and fluorescence intensity was monitored at 2‐min intervals for 60 min. Changes in the fluorescence ratio (340/380 nm) were used to infer alterations in intracellular Ca²⁺ levels following ionomycin treatment.

### 2.11 Assessment of Bacterial Membrane Potential

The fluorescent probe DiBAC4(3) was utilized to investigate the membrane depolarization activity of ionomycin (Abouelkhair et al. 2025; Alam et al. 2015). A log‐phase culture of *C. difficile* ATCC 43255 was centrifuged and washed twice with pre‐reduced PBS and resuspended to an OD_600_ of ~1. Ionomycin (3× MIC) and comparator agents (daptomycin (5× MIC) as a calcium‐dependent positive control; and VAN and FDX as calcium‐independent negative controls) were added to the bacterial suspensions in the presence or absence of CaCl₂ (100 mM). Cultures were incubated anaerobically at 37°C for 3 h, followed by staining with DiBAC4(3) (5 µM) for 30 min in the dark. Following an anaerobic incubation period of 30 min in the dark, the samples were centrifuged at 6000 rpm for 10 min in order to remove the dye. The fluorescence of DiBAC4(3) was detected using a BioTek Synergy H1 Hybrid plate reader with excitation and emission wavelengths of 493 and 516, respectively. Increased fluorescence intensity was interpreted as indicative of membrane depolarization.

### 2.12 Calcium Chelation Assay

To further confirm the mechanism of action of ionomycin's anti‐*C. difficile* activity, *C. difficile* ATCC 43255 was exposed to ionomycin in the presence of the calcium chelator, EGTA at varying concentrations of (400, 800, and 1000 μg/mL) and the MICs of ionomycin were determined as described above.

### 2.13 ATP Leakage Assay

The effect of calcium chelation on ionomycin‐induced membrane damage was assessed by quantifying extracellular ATP release using the BacTiter‐Glo Microbial Cell Viability Assay (Promega), as described previously (Abouelkhair et al. 2025; Roshan et al. 2019; Stolz et al. 2026). Log‐phase cultures of *C. difficile* ATCC 43255 (OD₆₀₀ ~1) were treated with ionomycin (5× MIC) in the presence of EGTA (1000 µg/mL) and incubated anaerobically at 37°C for 3 h. Following incubation, cultures were centrifuged at 10,000 × *g* for 10 min, and supernatants were collected. The luminescent signal corresponding to extracellular ATP levels was measured in the supernatants according to the manufacturer's protocol. Increased extracellular ATP levels were interpreted as indicative of membrane disruption and leakage.

### 2.14 Statistical Analysis

The statistical analysis was conducted using GraphPad Prism version 10 for Windows (GraphPad Software, La Jolla, California). The experiments were performed at least two times each containing the biological replicates. ANOVA was performed for statistical analysis as it provides a statistically robust and widely accepted method for evaluating treatment effects. ANOVA was performed on data sampled from a normal or lognormal distribution. The null hypothesis is that the populations from which the values were sampled all have the same geometric mean. Furthermore, the analysis assumes that the standard deviations/geometric standard deviations of those populations are equal. GraphPad Prism tests this assumption using two tests, the Brown‐Forsythe test and Bartlett's test.

## 3 Results and Discussion

### 3.1 Antibacterial Activity of Ionomycin Against *C. difficile*

**Figure 1:**
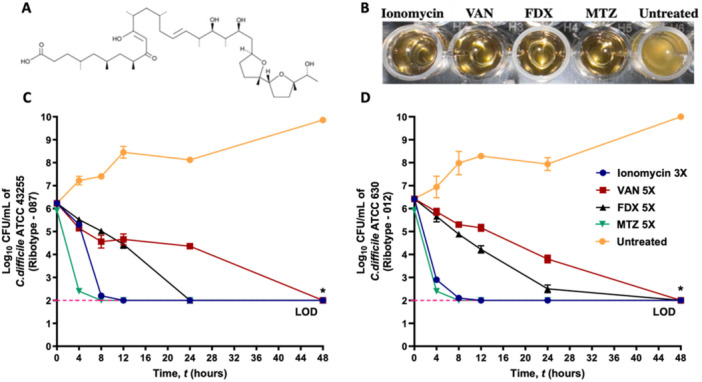
Anti‐*C. difficile* activity of ionomycin: (A) Chemical structure of ionomycin. (B) Inhibition of *C. difficile* growth by ionomycin at 3× MIC compared with comparator antibiotics (VAN, FDX, and MTZ) tested at 5× MIC. The MIC of MTZ against the tested strain ranged from 0.06 to 0.125 µg/mL. (C,D) Time–kill kinetics of ionomycin and comparator antibiotics against *C. difficile* ATCC 43255 (C) and ATCC 630 (D). DMSO (solvent) served as the growth control. Bacterial counts are expressed as mean log₁₀ CFU/mL ± standard deviation at each time point. Statistical significance was determined using two‐way ANOVA followed by Dunnett's post hoc test for multiple comparisons. An asterisk (*) denotes a statistically significant difference (*p* < 0.05) compared with the negative control. All experiments were performed in triplicate (three independent biological replicates).

The microbial metabolite library (HY‐L084), consisting of 527 compounds, was screened against *C. difficile* ATCC BAA‐1870 at a concentration of 16 μM (Abouelkhair and Seleem 2024). Following the exclusion of antibacterial agents, ionomycin (Figure 1A) was selected for further investigation against a panel of *C. difficile* strains. The anti*‐C. difficile* activity of ionomycin was evaluated against a panel of 30 clinical *C. difficile* isolates, including the most common ribotypes (027, 002, 087, 020, 001, 012, and 106) (Yadlapati et al. 2021) (Table 1). Ionomycin inhibited the growth of all clinical isolates at concentrations ranging from 0.5 to 2 μg/mL. It inhibited 50% of the tested isolates (MIC_50_) at a concentration of 1 μg/mL and inhibited 90% of the isolates (MIC_90_) at a concentration of 2 μg/mL (Table 1). Remarkably, ionomycin's MIC values were comparable to the MIC values of VAN, which showed MIC_50_ and MIC_90_ values of 0.5 and 1 μg/mL, respectively. While FDX exhibited MIC_50_ and MIC_90_ values of 0.015 and 0.06 μg/mL, respectively (Table 1). Importantly, ionomycin displayed consistent activity against hypervirulent NAP1/027 isolates, showing equal or superior potency compared with VAN. To further define its killing profile, the MBCs of ionomycin were determined for all isolates. The MBC values were equal to or only one dilution higher than the MICs, indicating a primarily bactericidal mode of action. This pattern paralleled that observed for VAN and FDX (Table 1). Collectively, these data establish ionomycin as a potent anti‐*C. difficile* agent with inhibitory activity comparable to current first‐line CDI therapeutics. Maintaining the same range of potency across multiple *C. difficile* ribotypes, including epidemic 027 and 106 lineages, suggests robustness against strain‐specific variability, a critical feature for potential therapeutic translation.

**Table 1:** MICs and MBCs (µg/mL) of ionomycin and control antibiotics against *C. difficile* strains.

*C. difficile* strains	Ionomycin		Control antibiotics			
			VAN^a^		FDX^b^	
	MIC	MBC	MIC	MBC	MIC	MBC
NR‐ 49279 (Ribotype ‐ 027)	1	1	0.25	0.5	0.03	0.06
NR‐ 49281 (Ribotype ‐ 027)	1	1	0.5	0.5	0.015	0.06
NR‐ 49283 (Ribotype ‐ 027)	2	2	0.25	0.5	0.03	0.06
NR‐ 49284 (Ribotype ‐ 027)	2	2	0.25	0.5	0.03	0.06
NR‐ 49288 (Ribotype ‐ 027)	1	1	0.25	0.5	0.015	0.015
NR‐ 49291 (Ribotype ‐ 027)	1	2	1	1	0.015	0.015
NR‐ 49303 (Ribotype ‐ 020)	1	2	0.5	1	0.06	0.06
NR‐ 49307 (Ribotype ‐ 002)	1	1	0.25	0.5	0.015	0.015
NR‐ 49309 (Ribotype ‐ 002)	1	1	0.5	1	≤ 0.008	0.008
NR‐ 49313 (Ribotype ‐ 017)	1	2	0.5	1	≤ 0.008	≤ 0.008
NR‐ 49317 (Ribotype ‐ 024)	1	1	0.5	0.5	0.03	0.06
NR‐ 49318 (Ribotype ‐ 106)	2	2	0.25	0.5	0.06	0.06
CD‐2 (Ribotype ‐ 056)	1	1	0.5	0.5	0.06	0.125
CD‐3 (Ribotype ‐ 015)	1	1	0.5	1	0.06	0.125
CD‐4 (Ribotype ‐ 002)	1	2	0.5	0.5	0.06	0.06
CD‐5 (Ribotype ‐ 027)	1	2	1	1	0.06	0.06
CD‐7 (Ribotype ‐ 020)	1	1	0.5	0.5	≤ 0.008	≤ 0.008
CD‐8 (Ribotype ‐ 002)	1	1	0.5	1	0.015	0.03
CD‐9 (Ribotype ‐ 019)	1	1	0.5	0.5	≤ 0.008	≤ 0.008
CD‐16 (Ribotype ‐ 054)	2	2	0.25	0.5	0.06	0.125
CD‐17 (Ribotype ‐ 078)	1	1	0.25	0.5	≤ 0.008	≤ 0.008
CD‐18 (Ribotype ‐ 002)	1	1	1	2	0.06	0.125
CD‐19 (Ribotype ‐ 106)	1	1	0.5	1	0.015	0.03
CD‐20 (Ribotype ‐ 015)	2	2	1	2	≤ 0.008	≤ 0.008
CD‐25 (Ribotype ‐ 014)	1	1	0.5	0.5	0.015	0.015
CD‐27 (Ribotype ‐ 106)	2	2	0.25	0.5	0.015	0.06
ATCC 630 (Ribotype ‐ 012)	0.5	1	0.25	0.5	≤ 0.008	≤ 0.008
ATCC 43255 (Ribotype ‐ 087)	1	1	0.25	0.5	0.03	0.06
ATCC BAA‐1870 (Ribotype ‐ 027)	1	2	1	1	0.015	0.015
ATCC 9689 (Ribotype ‐ 001)	1	2	0.25	0.5	≤ 0.008	≤ 0.008
**MIC**_**50**_	**1**		**0.5**		**0.015**	
**MIC**_**90**_	**2**		**1**		**0.06**	

*Note:* The bold text was used to distinguish the MIC_50_ and MIC_90_ values calculated from all tested isolates from the MIC values of individual isolates in the table.

^a^ Vancomycin.

^b^ Fidaxomicin.

### 3.2 Killing Kinetics of Ionomycin

To confirm the bactericidal activity of ionomycin against *C. difficile*, a time‐kill kinetic assay was performed using high inoculum of *C. difficile* ATCC 43255 and ATCC 630. As presented in Figure 1, ionomycin (3× MIC) produced a rapid decline in viable bacterial counts. Within 4 h, ionomycin reduced the bacterial burden of *C. difficile* ATCC 43255 by approximately 1 log₁₀ CFU/mL (Figure 1C) and that of ATCC 630 by over 3 log₁₀ CFU/mL (Figure 1D). Complete eradication of both strains occurred by 8 h, corresponding to a reduction below the assay detection limit (100 CFU/mL). For comparison, MTZ (5× MIC) exhibited a similar bactericidal pattern, achieving > 3 log₁₀ CFU/mL reduction within 8 h, consistent with previous findings (Kumar et al. 2014). In contrast, VAN and FDX (5× MIC) displayed significantly slower killing kinetics. VAN achieved only a 2 log₁₀ CFU/mL reduction after 24 h and required 48 h for complete bacterial clearance against both strains. FDX reduced bacterial counts more rapidly than VAN but still required 24 h for total eradication (Figure 1C and D), consistent with previous reports (Tyrrell et al. 2006). The rapid killing kinetics of ionomycin are particularly noteworthy, as swift bactericidal action is advantageous for treating *C. difficile* infections by rapidly decreasing toxin‐producing vegetative cells, limiting disease progression, and minimizing the emergence of resistant subpopulations (Stratton 2003).

### 3.3 Cytotoxicity of Ionomycin

**Figure 2:**
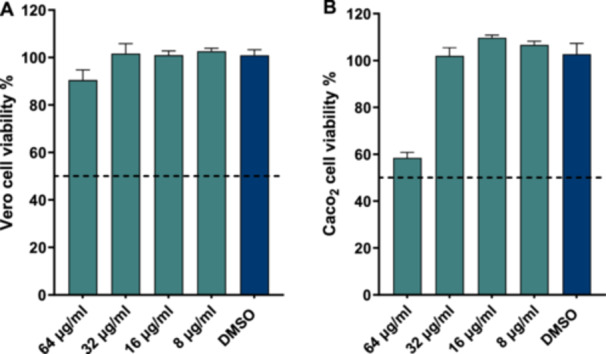
Cytotoxicity assessment of ionomycin in mammalian cell lines. Cytotoxicity of ionomycin was evaluated in African green monkey kidney (Vero) cells (A) and human colorectal epithelial (Caco‐2) cells (B) using the MTS [3‐(4,5‐dimethylthiazol‐2‐yl)‐5‐(3‐carboxymethoxyphenyl)‐2‐(4‐sulfophenyl)‐2H‐tetrazolium] assay following 24 h of exposure. Cell viability is expressed as the percentage of absorbance relative to the DMSO vehicle control. Ionomycin did not significantly reduce cell viability at concentrations up to 64 µg/mL in either cell line. DMSO served as the negative control.

To evaluate the safety window of ionomycin, its cytotoxicity was assessed against African green monkey kidney cells (Vero) (Figure 2A) and human colorectal epithelial cells (Caco‐2) (Figure 2B). Following 24 h of exposure, ionomycin did not significantly reduce cell viability compared with the vehicle control (DMSO) at concentrations up to 64 µg/mL in both cell lines (Figure 2). Accordingly, the CC₅₀ value (concentraton of the test agent that results in 50% cell viability) for ionomycin exceeded 64 µg/mL, corresponding to more than 64‐fold higher than the MIC of ionomycin against *C. difficile*. These findings indicate a wide in vitro therapeutic window and support that the antibacterial activity of ionomycin at the MIC is not attributable to nonspecific cytotoxicity toward mammalian cells.

### 3.4 Ionomycin Inhibits *C. difficile* Toxin Production

**Figure 3:**
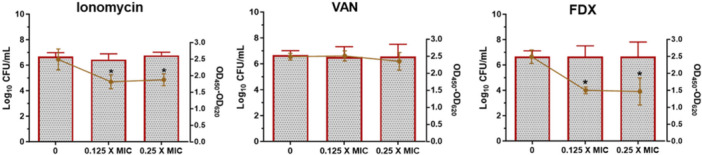
Toxin inhibition activity of ionomycin against *C. difficile*. Ionomycin, VAN, and FDX (at 0.125 × and 0.25 × MIC) were incubated with the *C. difficile* ATCC 43255. Bacterial viability (bars, left axis) was determined by CFU enumeration on pre‐reduced BHIS agar. Toxin levels (lines, right axis) were quantified from culture supernatants using ELISA and expressed as absorbance values (OD₄₅₀–OD₆₂₀). Data are presented as mean ± SD from three biological replicates. Statistical analysis was performed using one‐way ANOVA with Dunnett's post hoc test. *p* < 0.05 compared with untreated control.

Following confirmation of ionomycin's potent bactericidal activity, we next evaluated its effect on the two principal virulence factors of *C. difficile*; toxin production and spore formation. Toxin production is the primary mediator of *C. difficile* pathogenesis, driving severe intestinal inflammation, epithelial disruption, and colonic necrosis through the cytotoxic actions of toxins A (TcdA) and B (TcdB) (Davies et al. 2011; Chumbler et al. 2012). Agents that suppress toxin synthesis therefore hold substantial therapeutic promise by attenuating disease severity independently of bacterial killing. As shown in Figure 3, ionomycin at subinhibitory concentrations (0.125× and 0.25× MIC) reduced total toxin production by approximately 27% relative to untreated controls. Importantly, CFU enumeration demonstrated no significant reduction in bacterial viability under these conditions, confirming that toxin suppression was not a secondary effect of impaired bacterial growth. The toxin‐inhibitory activity of ionomycin was comparable to that of fidaxomicin (FDX), a known suppressor of *C. difficile* toxin production (Babakhani et al. 2013), which achieved 39.7% and 41.3% reductions in total toxin production at 0.125× and 0.25× MIC, respectively. In contrast, VAN as expected, failed to reduce toxin levels, consistent with previous reports (Babakhani et al. 2012b; Bassères et al. 2016; Roshan et al. 2018; AbdelKhalek et al. 2019). These findings demonstrate that ionomycin, at subinhibitory concentrations, effectively attenuates *C. difficile* virulence by reducing toxin production, a key determinant of disease pathology. Collectively, these results highlight ionomycin as a unique anti‐*C. difficile* agent that combines rapid bactericidal activity with a toxin‐suppressive effect, a dual property not observed with standard‐of‐care agents such as VAN.

### 3.5 Ionomycin Inhibits *C. difficile* Spore Formation

**Figure 4:**
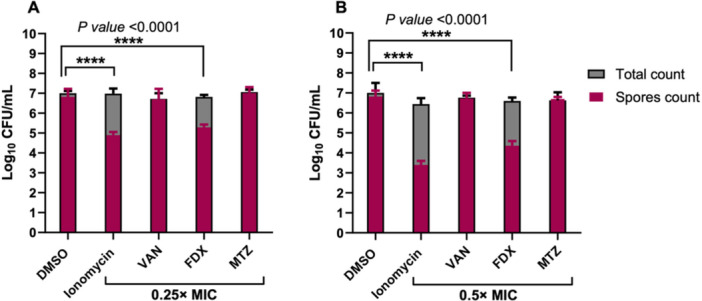
Spore formation inhibitory activity of ionomycin and control antibiotics against *C. difficile* ATCC 43255. Drugs (0.25× (A) and 0.5× MIC (B)) were incubated with bacteria for 6 days. DMSO was included as a negative control. Total counts were determined by serial dilution and plating onto BHIS agar plates supplemented with 0.1% taurocholic acid. Spores' counts were determined after heating at 65°C for 40 min followed by serial dilution and plating onto pre‐reduced BHIS agar plates supplemented with 0.1% taurocholic acid. The data are presented as average (triplicate samples for each treatment) ± standard deviation. The data were analyzed via two‐way ANOVA with post hoc Dunnett's test for multiple comparisons. An asterisk (*) indicates a statistically significant difference (*p* < 0.0001) between treatment with test agents as compared to the negative control (DMSO). The experiment was performed in triplicates (three biological replicates for each).

While toxin production drives the acute manifestations of CDI, spore formation underlies *C. difficile* transmission, persistence, and recurrence (Vedantam et al. 2012; Gerding et al. 2008). Given ionomycin's observed inhibition of *C. difficile* toxin production, we next investigated its ability to interfere with the sporulation process. As shown in Figure 4, ionomycin significantly reduced spore formation in a concentration‐dependent manner. At subinhibitory concentrations (0.25× and 0.5× MIC), ionomycin reduced spore counts by 1.92 log₁₀ and 3.42 log₁₀, respectively, following 6 days of anaerobic incubation compared with untreated controls. This reduction was substantially greater than that observed with FDX, which decreased spore formation by 1.53 log₁₀ and 2.46 log₁₀ at 0.25× and 0.5× MIC, respectively. In contrast, VAN and MTZ did not produce a measurable decrease in spore formation, consistent with prior studies (Babakhani et al. 2012a). The ability of ionomycin to inhibit spore formation is particularly significant, as spores represent the primary source of environmental persistence and recurrent CDI. Agents capable of targeting both vegetative cells and spores remain rare, and the observed dual activity of ionomycin suggests its potential to reduce disease relapse and transmission. Overall, these findings establish ionomycin as a multifunctional anti‐*C. difficile* agent that not only exerts rapid bactericidal activity but also attenuates key virulence factors, including toxin production and sporulation. This dual inhibition may translate to superior clinical outcomes by simultaneously suppressing disease progression and preventing recurrence.

### 3.6 Ionomycin Reduces Spore Outgrowth During Germination

Spore germination is a critical early event in the pathogenesis of *C. difficile*. Dormant spores sense host‐derived bile acids, such as taurocholate, glycocholate, and deoxycholate, along with l‐glycine as a co‐germinant, triggering the transition to metabolically active vegetative cells capable of colonization, toxin production, and disease initiation (Sorg and Sonenshein 2008; Howerton et al. 2013). Therefore, identifying agents that prevent or eliminate germinating spore outgrowth is an essential strategy for effective CDI treatment.

Unlike prior spore outgrowth studies that focus primarily on early germination events within 24–48 h (Chilton et al. 2016; Allen et al. 2013), we extended the incubation period up to 14 days to assess the durability of antibacterial activity and the potential to suppress delayed outgrowth, a process that is clinically relevant to CDI recurrence. Notably, CDI relapse frequently occurs 8–10 days after cessation of therapy, driven by surviving spores that subsequently germinate and re‐establish infection. Thus, this extended observation window was designed to model long‐term suppression of spore‐driven regrowth following antimicrobial exposure.

**Figure 5:**
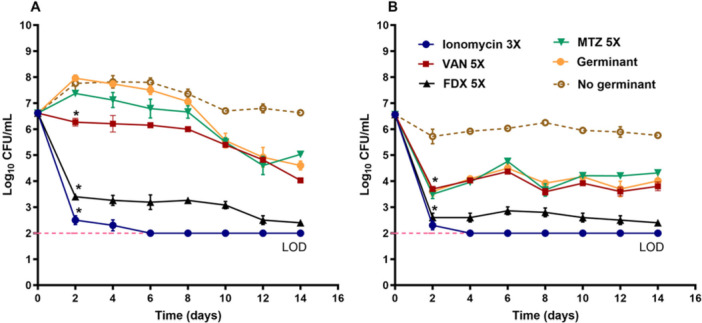
Measuring outgrowth of *C. difficile* spores during ionomycin treatment. *C. difficile* spores were incubated with either ionomycin (3× MIC) or the control antibiotics (VAN, FDX, and MTZ, at 5× MIC) for 14 days in BHIS broth supplemented with 0.1% taurocholic acid and 10 mM glycine (germinant). Spores treated with DMSO (with and without germinant) served as controls. (A) Log_10_ CFU/mL of the total counts (average ± standard deviation). (B) Log_10_ CFU/mL of the spore counts after heating at 65° C for 40 min. and plating onto BHIS agar plates containing 0.1% taurocholic acid. The data were analyzed via a two‐way ANOVA with post‐hoc Dunnett's test for multiple comparisons. An asterisk (*) indicates a statistically significant difference (*p* < 0.05) between treatment with test agents as compared to the negative control. The experiment was performed in triplicates.

Under germination‐permissive conditions, ionomycin demonstrated potent and sustained activity against *C. difficile* spore outgrowth. Treatment with ionomycin at 3× MIC reduced total viable counts by approximately 4 log₁₀ CFU by day 2 and resulted in complete eradication of both vegetative cells and spores, falling below the limit of detection by day 4 (Figure 5A). This rapid and complete clearance was comparable to fidaxomicin (FDX) at 5× MIC, which achieved an approximate 3.5 log₁₀ CFU reduction but failed to fully eliminate the population even after 14 days of incubation. In contrast, vancomycin (VAN) and metronidazole (MTZ) showed no reduction beyond germinant‐only controls, indicating a lack of activity against germinating spores or subsequent vegetative outgrowth.

Assessment of heat‐resistant spores further confirmed these findings. After heat treatment, ionomycin and FDX each produced a ~ 4log₁₀ reduction in spore counts, consistent with their effects on total viable cells (Figure 5B). Conversely, VAN and MTZ treatment resulted in an initial ~3 log₁₀ reduction by day 2, reflecting the loss of vegetative cells, which are heat‐sensitive, but no further decline in the viable spore population thereafter. This plateau indicates that neither VAN nor MTZ exerted sporicidal activity or inhibited germination under the tested conditions, consistent with previous studies (Chilton et al. 2016). Together, these results demonstrate that ionomycin not only suppresses spore outgrowth but also eliminates heat‐resistant spores.

To evaluate whether ionomycin could also limit toxin production following germination, culture supernatants collected at 2, 8, and 14 days were filtered and applied to Vero cell monolayers to assess cytotoxicity. Consistent with its potent sporicidal activity, ionomycin‐treated samples produced minimal toxin‐mediated cytotoxicity, maintaining ~90% Vero cell viability across all time points (Figure S1). This low cytotoxicity reflects the dual action of ionomycin in both eliminating germinating spores and suppressing toxin production from any residual vegetative cells. Ionomycin's protective effect was comparable to that of FDX, which is known to inhibit toxin synthesis in vegetative *C. difficile* (Chilton et al. 2016; Babakhani et al. 2012b). Conversely, supernatants from VAN‐ and MTZ‐treated cultures caused 50%–90% cytotoxicity, reflecting their inability to inhibit germination or toxin production.

### 3.7 Evaluation of the Activity of Ionomycin Against the Normal Intestinal Microbiota

Antibiotic‐mediated disruption of the intestinal microbiota is a major predisposing factor for CDI. The currently available anti‐CDI agents, such as VAN, MTZ, and even FDX can disturb the delicate balance of the gut ecosystem, leading to dysbiosis, reduced colonization resistance, and elevated recurrence rates (Yamaguchi et al. 2020a). Therefore, the development of narrow‐spectrum agents with selective activity against *C. difficile* and minimal collateral impact on the beneficial intestinal flora remains a crucial goal in CDI therapy. To evaluate the selectivity of ionomycin, we assessed its antibacterial activity against representative bacteria that comprise the human normal gut microbiome including *Bifidobacterium, Bacteroides*, and *Lactobacillus* species. The MICs of ionomycin and reference anti‐*C. difficile* agents (VAN, FDX, and MTZ) are summarized in Table 2. Ionomycin exhibited minimal inhibitory activity against all tested commensal species, with MIC values of > 256 µg/mL (Table 2). In contrast, the control antibiotics, VAN, FDX, and MTZ, demonstrated a potent activity against most intestinal microbiota strains tested, as reported previously (Yamaguchi et al. 2020b; Qian et al. 2023). VAN inhibited the growth of *Bifidobacterium* species at concentrations ≤ 2 µg/mL and demonstrated moderate activity against *Bacteroides* species (MIC = 16–128 µg/mL). MTZ exhibited potent inhibition of both *Bifidobacterium* and *Bacteroides* isolates, with MICs ranging from ≤ 2 µg/mL to 8 µg/mL. FDX, while more selective than VAN or MTZ, still suppressed *Bifidobacterium* growth at ≤ 2 µg/mL and exhibited moderate activity against *Lactobacillus* species (MIC = 2–4 µg/mL). Collectively, these findings demonstrate that ionomycin exerts minimal activity against key constituents of the beneficial gut microbiota, contrasting sharply with the broad‐spectrum effects of current standard‐of‐care agents. This high selectivity profile suggests that ionomycin could offer an important therapeutic advantage by preserving microbiome integrity during CDI treatment and potentially reducing the risk of post‐treatment recurrence.

**Table 2:** MICs (µg/mL) of ionomycin and control anti‐*C. difficile* drugs against representative human gut microbiota strains.

Representative gut microbiota strains	MICs (μg/mL)			
	Ionomycin	VAN	FDX	MTZ
*Bifidobacterium breve* HM‐856	> 256	≤ 2	≤ 2	8
*Bacteroides finegoldii* HM‐727	> 256	64	> 256	≤ 2
*Bacteroides stercoris* HM‐1036	> 256	128	> 256	≤ 2
*Bacteroides fragilis* HM‐20	> 256	16	128	≤ 2
*Bacteroides fragilis* HM‐710	> 256	128	> 256	≤ 2
*Bacteroides fragilis* HM‐714	> 256	32	> 256	≤ 2
*Bacteroides fragilis* HM‐ 711	> 256	32	> 256	≤ 2
*Bacteroides caccae* HM‐728	> 256	32	> 256	≤ 2
*Bacteroides dorei* HM‐29	> 256	32	> 256	≤ 2
*Lactobacillus gasseri* ATCC 19992	> 256	> 256	4	> 256
*Lacticaseibacillus rhamnosus* HM‐106	> 256	> 256	2	> 256

### 3.8 Extracellular Calcium Enhances the Anti‐*C. difficile* Activity of Ionomycin

Ionomycin, a well‐known calcium ionophore, facilitates the transmembrane transport of calcium ions, leading to elevated intracellular calcium concentrations within cells (Liu and Hermann 1978; Morgan and Jacob 1994). Calcium ions play a pivotal role in numerous cellular processes across all domains of life, serving as a ubiquitous signaling molecule that regulates functions such as enzyme activity, gene expression, and membrane stability (Dominguez 2004; Rosch et al. 2008). However, while eukaryotic calcium signaling has been extensively characterized, relatively little is known about the mechanisms of calcium homeostasis and signaling in prokaryotic systems. In bacteria, intracellular calcium levels are typically maintained at very low concentrations relative to the extracellular environment, creating a steep calcium gradient across the cell membrane that can profoundly affect cell viability (Gupta et al. 2017). This tight regulation is crucial for normal physiological function and survival. For instance, *Streptococcus pneumoniae* actively expels calcium to maintain homeostasis, so mutants lacking efficient calcium efflux mechanisms are unable to survive in high‐calcium environments, like the lung, due to calcium overload (Rosch et al. 2008; Flego et al. 1997).

**Figure 6:**
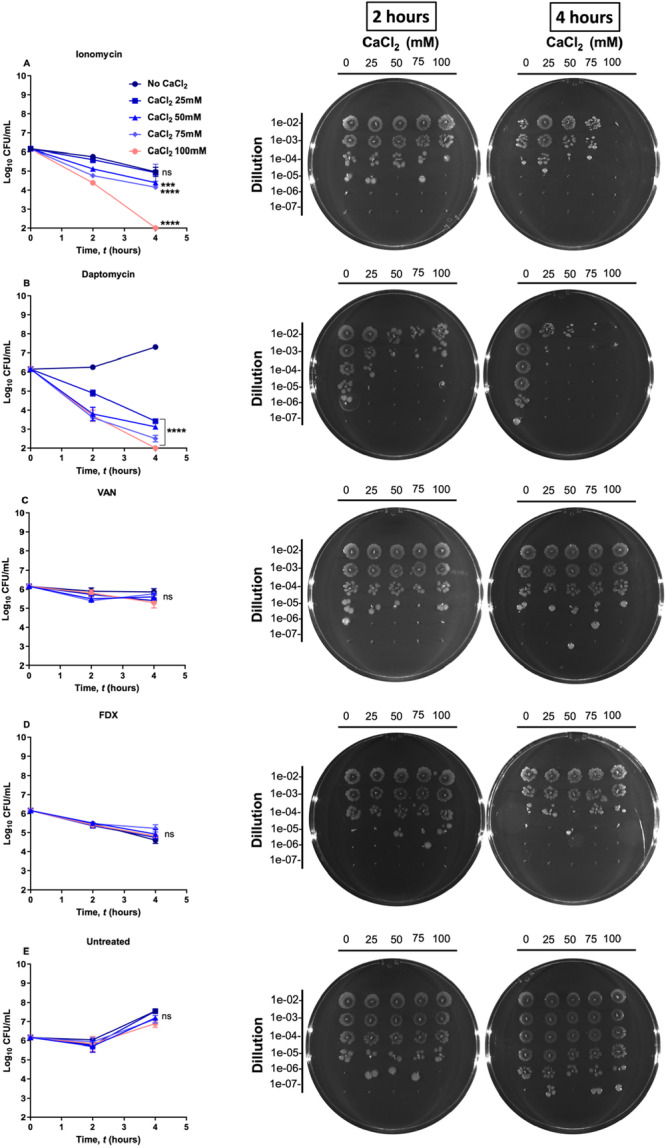
Effect of calcium supplementation on the anti‐*C. difficile* activity of ionomycin. *C. difficile* ATCC 43255 was treated with either ionomycin (3× MIC) or control antibiotics, VAN, FDX and daptomycin (5× MIC). Calcium was supplemented at the concentrations of 0, 25, 50, 75, and 100 mM. Aliquots were taken after 2 and 4 h, diluted and plated onto BHIS agar plates. The error bars represent standard deviation values for each test agent. The CFU data were analyzed via one‐way ANOVA with post hoc Dunnett's test for multiple comparisons. An asterisk (*) indicates a statistically significant difference (***, *p* = 0.0002, ****, *P* < 0.0001) between treatment with test agents, in the presence of different CaCl_2_ concentrations, as compared to the negative control (no CaCl_2_). The experiment was performed in triplicates (three biological replicates for each).

Despite the recognized importance of calcium regulation in other bacteria, little is known about calcium efflux or homeostatic mechanisms in *C. difficile*. To determine whether ionomycin exerts its potent anti‐*C. difficile* activity through disruption of calcium homeostasis, we investigated the relationship between extracellular calcium concentration and bacterial viability in *C. difficile* treated with ionomycin. Log‐phase cultures of *C. difficile* ATCC 43255 were exposed to ionomycin (3× MIC) in the presence of increasing concentrations of CaCl₂ (0, 25, 50, 75, and 100 mM). Viable cell counts were determined after 2 and 4 h of incubation. In the absence of calcium, ionomycin produced no significant reduction in bacterial counts. However, as extracellular calcium concentration increased, the bactericidal activity of ionomycin was markedly enhanced. In the presence of 50 and 75 mM CaCl₂, a ~ 0.5 log₁₀ CFU reduction was observed after 4 h, whereas with 100 mM CaCl₂, ionomycin completely eradicated the inoculum (~6 log₁₀ CFU reduction) within the same period (Figure 6A). As controls, daptomycin (a positive control known to cause membrane depolarization and is active in presence of calcium) (Figure 6B), VAN, (a cell wall synthesis inhibitor (Eubank et al. 2022)) (Figure 6C), and FDX (an RNA polymerase inhibitor (Zhanel et al. 2015)) (Figure 6D) were included. As expected, increasing extracellular calcium had no appreciable effect on the activity of VAN or FDX, and viable counts remained similar to those in calcium‐free conditions. In contrast, daptomycin exhibited a calcium‐dependent enhancement of activity. Collectively, these results suggest that the bactericidal effect of ionomycin is mediated by calcium influx and intracellular accumulation (Figure 6).

### 3.9 Ionomycin Drives Intracellular Calcium Accumulation in *C. difficile*

**Figure 7:**
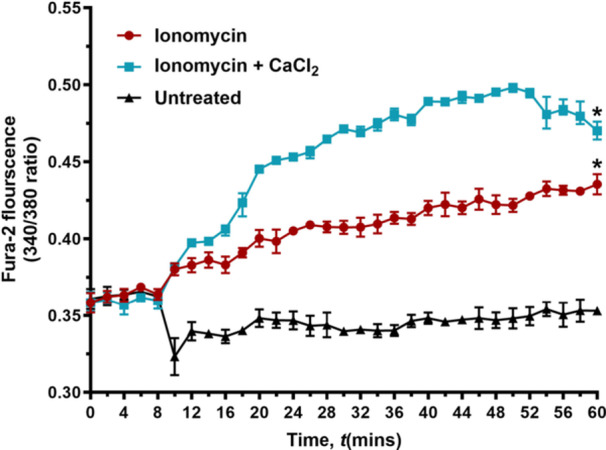
Ionomycin induces intracellular calcium accumulation in *C. difficile.* Intracellular calcium levels were quantified in *C. difficile* ATCC 43255 using Fura‐2 AM following treatment with ionomycin (5× MIC) in the presence or absence of 100 mM CaCl₂, and the 340/380 fluorescence ratios were determined for each treatment. The data were analyzed via a two‐way ANOVA with post‐hoc Dunnett's test for multiple comparisons. An asterisk (*) indicates a statistically significant difference (*p* < 0.05) between treatment with test agents as compared to the negative control.

To further evaluate whether ionomycin mediates its anti‐*C. difficile* activity through disruption of calcium homeostasis, we quantified the intracellular calcium levels in *C. difficile* following exposure to ionomycin, with or without extracellular calcium supplementation. In untreated cells, the fluorescence ratio (340/380 nm) remained stable, indicating basal intracellular calcium levels. In contrast, ionomycin alone produced a measurable and time‐dependent increase in intracellular calcium, with the 340/380 ratio rising from 0.37 at 8 min to 0.43 after 60 min of incubation (Figure 7). This modest but consistent elevation confirms that ionomycin facilitates calcium influx into *C. difficile* even under standard assay conditions. Importantly, supplementation with 100 mM CaCl₂ in combination with ionomycin resulted in a substantially greater increase in intracellular calcium, with the 340/380 ratio rising from 0.37 to 0.50 over the same period. This amplified signal reflects enhanced calcium entry driven by ionomycin in the presence of an elevated extracellular calcium gradient.

Collectively, these findings demonstrate that ionomycin disrupts *C. difficile* calcium homeostasis by promoting intracellular calcium accumulation, and that this effect is strongly potentiated by extracellular calcium availability. The correlation between elevated intracellular calcium and the observed bactericidal activity supports a model in which ionomycin‐mediated calcium overload contributes directly to cell death, consistent with the calcium‐dependent killing observed in our viability assays.

### 3.10 Ionomycin Disrupts the Membrane Potential in *C. difficile* and Calcium Supplementation Enhances This Effect

**Figure 8:**
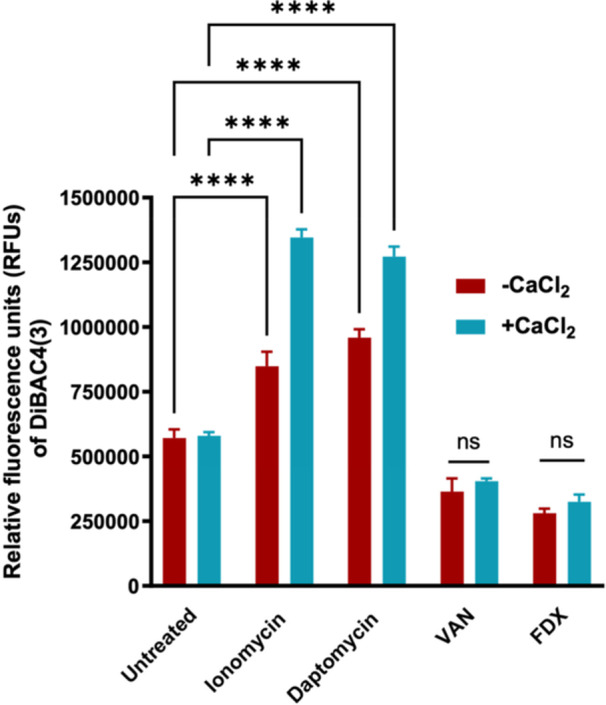
Ionomycin disrupts the membrane potential in *C. difficile. C. difficile* ATCC 43255 was treated with ionomycin (alone and in combination with 100 mM CaCl_2_ for 3 h). Daptomycin was included as a positive control drug, and VAN and FDX were included as negative control drugs. The fluorescent dye DiBAC4(3) was added and the fluorescence intensity of DiBAC4(3) after treatment with test agents (alone and in combination with CaCl_2_) was monitored using a BioTek Synergy H1 Hybrid Microplate Reader with excitation and emission wavelengths of 493 and 516, respectively. The data are presented as average (triplicate samples for each treatment) ± standard deviation. Statistical analysis was performed using a two‐way ANOVA with post hoc Dunnett's test for multiple comparisons (*p* < 0.0001).

Ionophores are known to disrupt the proton motive force, a key driver of bacterial energy metabolism, by facilitating uncontrolled ion flux across cellular membranes (Gooyit and Janda 2016). Previous studies have also shown that elevated intracellular calcium levels can hyperpolarize membrane potentials, including those of mitochondria in eukaryotic systems (Marshall et al. 2015). Given that ionomycin demonstrated ability to increase intracellular calcium accumulation, we next assessed its effect on *C. difficile* membrane potential using the voltage‐sensitive fluoroprobe DiBAC4(3) (Arthithanyaroj et al. 2021; Alam et al. 2015). Within 3 h of exposure, ionomycin induced a clear depolarization of the *C. difficile* membrane, as evidenced by a significant increase in DiBAC4(3) fluorescence (Figure 8). Notably, supplementation with extracellular calcium further amplified this signal, indicating an enhanced disruption of membrane potential under conditions favoring calcium influx. This effect parallels the activity of daptomycin, a control antibiotic known to perturb bacterial membrane potential through calcium‐dependent mechanisms. As expected, VAN and FDX produced fluorescence levels comparable to untreated controls, consistent with their non‐membrane‐targeting mechanisms of action. Altogether, these findings suggest that ionomycin promotes intracellular calcium overload that subsequently collapses membrane potential, ultimately contributing to bacterial cell death.

### 3.11 Chelation of Calcium Abolishes Ionomycin's Activity and Reduces Membrane Damage

**Figure 9:**
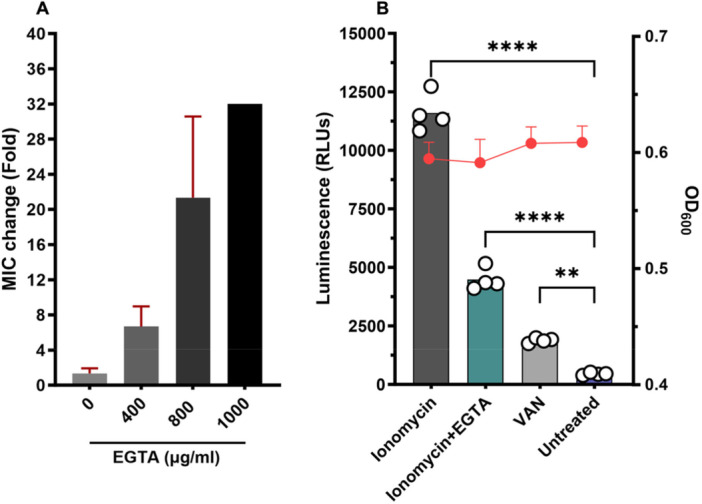
The impact of calcium chelation on ionomycin's anti‐*C. difficile* activity. (A) *C. difficile* ATCC 43255 was exposed to ionomycin in the presence of increasing concentrations of EGTA (0, 400, 800, and 1000 µg/mL), and the change in the MIC of ionomycin against *C. difficile*, relative to its MIC in the absence of EGTA, was determined. (B) Extracellular ATP levels of *C. difficile* ATCC 43255 that was exposed to ionomycin or VAN (at 5 × MIC) and the relative percentage of extracellular ATP (compared to the control (DMSO) was calculated and plotted. ATP measurements were normalized to the corresponding OD₆₀₀ values measured after the 3 h incubation period for each test agent to rule out the ATP leakage effect from total cell lysis.

To further confirm the role of calcium in ionomycin‐mediated killing, *C. difficile* ATCC 43255 was exposed to ionomycin in the presence of increasing concentrations of the calcium‐specific chelator EGTA (0, 400, 800, and 1000 µg/mL). The MIC of ionomycin increased proportionally with EGTA concentration, by 8‐, 16‐, and 32‐fold with 400, 800, and 1000 µg/mL EGTA, respectively (Figure 9A). This pattern is consistent with the known calcium‐dependent activity of daptomycin, which similarly lost potency upon adding calcium. In contrast, the MICs of VAN and FDX remained unchanged in the presence of EGTA. This calcium‐dependent shift in susceptibility provides strong evidence that ionomycin's anti‐*C. difficile* activity is mediated through intracellular calcium accumulation, ultimately leading to bacterial cell death.

In addition, the impact of calcium chelation on ionomycin‐induced membrane damage was assessed through ATP leakage assays. Treatment with ionomycin alone (5× MIC) significantly increased extracellular ATP (~11,000 RLUs) compared to untreated controls (DMSO), reflecting severe membrane disruption. Co‐treatment with EGTA markedly reduced ATP leakage (~5000 RLUs), indicating that calcium availability is essential for ionomycin‐mediated membrane permeabilization (Figure 9B).

In conclusion, this study identifies ionomycin as a highly potent and selective inhibitor of *C. difficile*, exhibiting multifaceted antimicrobial activities that target key drivers of CDI pathogenesis and recurrence. Ionomycin demonstrated robust bactericidal activity, significantly suppressed toxin production and spore formation, and effectively inhibited spore germination and subsequent toxin expression, properties that compare favorably to, and in some aspects exceed, those of current standard‐of‐care therapies such as vancomycin and fidaxomicin. Its selective activity against *C. difficile* relative to representative commensal gut bacteria, together with its unique calcium‐dependent mechanism involving membrane potential disruption, underscores its promise as a targeted anti‐CDI agent. Collectively, this work provides strong mechanistic and preclinical evidence supporting ionomycin as a compelling lead compound for the development of next‐generation, microbiota‐sparing CDI therapeutics.

Nevertheless, there are two limitations present in this study that would need to be addressed as part of a future investigation. First, assessment of ionomycin's anti‐commensal activity against selected intestinal commensals does not fully capture the complexity of the gut microbiota. Future studies employing ex vivo fecal community models, microbiome sequencing, and in vivo microbiota analyses will be important to more comprehensively evaluate the impact of ionomycin on gut microbial composition. Second, further investigation into pharmacokinetics, in vivo efficacy, and structural optimization will be critical to advancing ionomycin toward clinical development.

## Authors Contributions

**Ahmed Abouelkhair:** experimental design, data collection, figure preparation, and primary manuscript drafting. **Dr. Nader Abutaleb:** data analysis and substantial manuscript editing. **Prof. Mohamed Seleem:** conceptualization, supervision, funding acquisition, critical manuscript revision, and project administration.

## Ethics Statement

The authors have nothing to report.

## Conflicts of Interest

The authors declare no conflicts of interest.

## Supporting information


**Figure S1:** Evaluation of Ionomycin's Ability to Inhibit *C. difficile* Spore Germination and control anticlostridial agents against *C. difficile* ATCC 43255. Vero cells cytotoxicity after challenge with the supernatants of spores incubated with test agents for 2 (A), 8 (B), and 14 days (C). Supernatants were collected, filtered, mixed with DMEM (1:9) and added Vero cell monolayers to determine the cytotoxic impact of toxins produced after germination of *C. difficile* spores. The cell rounding was analyzed by the confocal microscopy and the cell cytotoxicity (average ± standard deviation) was calculated. The data were analyzed via one‐way ANOVA with post hoc Dunnett's test for multiple comparisons (*P*< 0.0001). Asterisks indicate statistically significant difference between the data of cell viability for different treatment as compared to the negative control (spores with germinant). **Table S1:** Description of *C. difficile* strains used in the study. **Table S2:** Description of the gut microbiota strains used in the study.

## References

[mbo370269-bib-0001] AbdelKhalek, A. , N. S. Abutaleb , H. Mohammad , and M. N. Seleem . 2019. “Antibacterial and Antivirulence Activities of Auranofin Against *Clostridium difficile* .” International Journal of Antimicrobial Agents 53, no. 1: 54–62. 30273668 10.1016/j.ijantimicag.2018.09.018 PMC6475173

[mbo370269-bib-0002] Abouelkhair, A. A. , N. S. Abutaleb , and M. N. Seleem . 2025. “The Antiarrhythmic Drugs Dronedarone and Amiodarone Exhibit Potent In Vitro and In Vivo Activity Against *Clostridioides difficile* .” International Journal of Antimicrobial Agents 67, no. 1: 107649. https://www.sciencedirect.com/science/article/pii/S0924857925002043. 41161584 10.1016/j.ijantimicag.2025.107649 PMC12699489

[mbo370269-bib-0003] Abouelkhair, A. A. , and M. N. Seleem . 2024. “Exploring Novel Microbial Metabolites and Drugs for Inhibiting Clostridioides Difficile.” mSphere 9, no. 7: e00273‐24. 38940508 10.1128/msphere.00273-24 PMC11288027

[mbo370269-bib-0004] Abutaleb, N. S. , and M. N. Seleem . 2020a. “Auranofin, at Clinically Achievable Dose, Protects Mice and Prevents Recurrence From Clostridioides Difficile Infection.” Scientific Reports 10, no. 1: 7701. 32382070 10.1038/s41598-020-64882-9 PMC7206065

[mbo370269-bib-0005] Abutaleb, N. S. , and M. N. Seleem . 2020b. “Repurposing the Antiamoebic Drug Diiodohydroxyquinoline for Treatment of *Clostridioides difficile* Infections.” Antimicrobial Agents and Chemotherapy 64, no. 6. 10.1128/aac.02115-19. PMC7269495 32253206

[mbo370269-bib-0006] Alam, M. Z. , X. Wu , C. Mascio , L. Chesnel , and J. G. Hurdle . 2015. “Mode of Action and Bactericidal Properties of Surotomycin Against Growing and Nongrowing *Clostridium difficile* .” Antimicrobial Agents and Chemotherapy 59, no. 9: 5165–5170. 26055381 10.1128/AAC.01087-15 PMC4538476

[mbo370269-bib-0007] Allen, C. A. , F. Babakhani , P. Sears , L. Nguyen , and J. A. Sorg . 2013. “Both Fidaxomicin and Vancomycin Inhibit Outgrowth of *Clostridium difficile* Spores.” Antimicrobial Agents and Chemotherapy 57, no. 1: 664–667. 23147724 10.1128/AAC.01611-12 PMC3535933

[mbo370269-bib-0008] Ammara, A. , S. Giovannuzzi , A. Bonardi , et al. 2025. “Redesigning Oxazolidinones as Carbonic Anhydrase Inhibitors Against Vancomycin‐Resistant Enterococci.” European Journal of Medicinal Chemistry 291: 117620. 40267877 10.1016/j.ejmech.2025.117620 PMC12186203

[mbo370269-bib-0009] Antrim, A. 2022. “Experts Urge FDA to Speed Approval of New Treatments, Preventative Measures for Recurrent C. Diff.” *Pharmacy Times*.

[mbo370269-bib-0010] Arthithanyaroj, S. , S. Chankhamhaengdecha , U. Chaisri , R. Aunpad , and A. Aroonnual . 2021. “Effective Inhibition of *Clostridioides difficile* by the Novel Peptide CM‐A.” PLoS One 16, no. 9: e0257431. 34516580 10.1371/journal.pone.0257431 PMC8437281

[mbo370269-bib-0011] Babakhani, F. , L. Bouillaut , A. Gomez , P. Sears , L. Nguyen , and A. L. Sonenshein . 2012a. “Fidaxomicin Inhibits Spore Production in *Clostridium difficile* .” supplement, Clinical Infectious Diseases 55, no. Suppl 2: S162–S169. 22752866 10.1093/cid/cis453 PMC3388029

[mbo370269-bib-0012] Babakhani, F. , L. Bouillaut , P. Sears , C. Sims , A. Gomez , and A. L. Sonenshein . 2012b. “Fidaxomicin Inhibits Toxin Production in *Clostridium difficile* .” Journal of Antimicrobial Chemotherapy 68, no. 3: 515–522. 23208832 10.1093/jac/dks450

[mbo370269-bib-0013] Babakhani, F. , L. Bouillaut , P. Sears , C. Sims , A. Gomez , and A. L. Sonenshein . 2013. “Fidaxomicin Inhibits Toxin Production in *Clostridium difficile* .” Journal of Antimicrobial Chemotherapy 68, no. 3: 515–522. 23208832 10.1093/jac/dks450

[mbo370269-bib-0014] Barbut, F. , A. Richard , K. Hamadi , V. Chomette , B. Burghoffer , and J. C. Petit . 2000. “Epidemiology of Recurrences or Reinfections of *Clostridium difficile*‐Associated Diarrhea.” Journal of Clinical Microbiology 38, no. 6: 2386–2388. 10835010 10.1093/gao/9781884446054.article.t031141 PMC86814

[mbo370269-bib-0015] Bassères, E. , B. T. Endres , M. Khaleduzzaman , et al. 2016. “Impact on Toxin Production and Cell Morphology in *Clostridium difficile* by Ridinilazole (SMT19969), a Novel Treatment for C. Difficile Infection.” Journal of Antimicrobial Chemotherapy 71, no. 5: 1245–1251. 26895772 10.1093/jac/dkv498 PMC4830417

[mbo370269-bib-0016] Di Bella, S. , P. Ascenzi , S. Siarakas , N. Petrosillo , and A. Di Masi . 2016. “ *Clostridium difficile* Toxins A and B: Insights Into Pathogenic Properties and Extraintestinal Effects.” Toxins 8, no. 5: 134. 27153087 10.3390/toxins8050134 PMC4885049

[mbo370269-bib-0017] Castro‐Córdova, P. , P. Mora‐Uribe , R. Reyes‐Ramírez , et al. 2021. “Entry of Spores into Intestinal Epithelial Cells Contributes to Recurrence of *Clostridioides difficile* Infection.” Nature Communications 12, no. 1: 1140. 10.1038/s41467-021-21355-5 PMC7893008 33602902

[mbo370269-bib-0018] CDC ., CDC's 2022 Antibiotic Resistance Threats Report. 2022.

[mbo370269-bib-0019] Chilton, C. H. , G. S. Crowther , H. Ashwin , C. M. Longshaw , and M. H. Wilcox . 2016. “Association of Fidaxomicin With *C. difficile* Spores: Effects of Persistence on Subsequent Spore Recovery, Outgrowth and Toxin Production.” PLoS One 11, no. 8: e0161200. 27556739 10.1371/journal.pone.0161200 PMC4996525

[mbo370269-bib-0020] Chumbler, N. M. , M. A. Farrow , L. A. Lapierre , et al. 2012. “ *Clostridium difficile* Toxin B Causes Epithelial Cell Necrosis Through an Autoprocessing‐Independent Mechanism.” PLoS Pathogens 8, no. 12: e1003072. 23236283 10.1371/journal.ppat.1003072 PMC3516567

[mbo370269-bib-0021] Clementi, E. A. , et al. 2014. “Monitoring Changes in Membrane Polarity, Membrane Integrity, and Intracellular Ion Concentrations in Streptococcus Pneumoniae Using Fluorescent Dyes.” Journal of Visualized Experiments: JoVE no. 84: e51008. 10.3791/51008. 24637356 PMC4124894

[mbo370269-bib-0022] Cole, S. , and T. Stahl . 2015. “Persistent and Recurrent *Clostridium difficile* Colitis.” Clinics in Colon and Rectal Surgery 28, no. 2: 065–069. 10.1055/s-0035-1547333 PMC4442717 26034401

[mbo370269-bib-0023] Cornely, O. A. , M. A. Miller , T. J. Louie , D. W. Crook , and S. L. Gorbach . 2012. “Treatment of First Recurrence of *Clostridium difficile* Infection: Fidaxomicin Versus Vancomycin.” Clinical Infectious Diseases 55, no. Suppl 2: S154–S161. 22752865 10.1093/cid/cis462 PMC3388030

[mbo370269-bib-0024] Davies, A. H. , A. K. Roberts , C. C. Shone , and K. R. Acharya . 2011. “Super Toxins From a Super Bug: Structure and Function of *Clostridium difficile* Toxins.” Biochemical Journal 436, no. 3: 517–526. 21615333 10.1042/BJ20110106

[mbo370269-bib-0025] Dominguez, D. C. 2004. “Calcium Signalling in Bacteria.” Molecular Microbiology 54, no. 2: 291–297. 15469503 10.1111/j.1365-2958.2004.04276.x

[mbo370269-bib-0026] Edwards, A. N. , and S. M. McBride . 2016. “Isolating and Purifying *Clostridium difficile* Spores.” Methods in Molecular Biology 1476: 117–128. 27507337 10.1007/978-1-4939-6361-4_9 PMC5017156

[mbo370269-bib-0027] Eubank, T. A. , A. J. Gonzales‐Luna , J. G. Hurdle , and K. W. Garey . 2022. “Genetic Mechanisms of Vancomycin Resistance in *Clostridioides difficile*: A Systematic Review.” Antibiotics (USSR) 11, no. 2: 258. 10.3390/antibiotics11020258 PMC8868222 35203860

[mbo370269-bib-0028] Feuerstadt, P. , N. Theriault , and G. Tillotson . 2023. “The Burden of CDI in the United States: A Multifactorial Challenge.” BMC Infectious Diseases 23, no. 1: 132. 36882700 10.1186/s12879-023-08096-0 PMC9990004

[mbo370269-bib-0029] Flego, D. , M. Pirhonen , H. Saarilahti , T. K. Palva , and E. T. Palva . 1997. “Control of Virulence Gene Expression by Plant Calcium in the Phytopathogen Erwinia Carotovora.” Molecular Microbiology 25, no. 5: 831–838. 9364909 10.1111/j.1365-2958.1997.mmi501.x

[mbo370269-bib-0030] Gerding, D. N. , S. Johnson , M. Rupnik , and K. Aktories . 2014. “Clostridium Difficile Binary Toxin CDT: Mechanism, Epidemiology, and Potential Clinical Importance.” Gut Microbes 5, no. 1: 15–27. 24253566 10.4161/gmic.26854 PMC4049931

[mbo370269-bib-0031] Gerding, D. N. , C. A. Muto , and R. C. Owens, Jr. 2008. “Measures to Control and Prevent *Clostridium difficile* Infection.” Clinical Infectious Diseases 46: S43–S49. 18177221 10.1086/521861

[mbo370269-bib-0032] Gooyit, M. , and K. D. Janda . 2016. “Reprofiled Anthelmintics Abate Hypervirulent Stationary‐Phase *Clostridium difficile* .” Scientific Reports 6, no. 1: 33642. 27633064 10.1038/srep33642 PMC5025651

[mbo370269-bib-0033] Gupta, H. K. , S. Shrivastava , and R. Sharma . 2017. “A Novel Calcium Uptake Transporter of Uncharacterized P‐Type ATPase Family Supplies Calcium for Cell Surface Integrity in *Mycobacterium smegmatis* .” mBio 8, no. 5. 10.1128/mbio.01388-17. PMC5615198 28951477

[mbo370269-bib-0034] Holly, K. J. , P. R. Tharra , N. S. Abutaleb , et al. 2025. “Hit‐to‐Lead Optimization of Acetazolamide‐Based Bacterial Carbonic Anhydrase Inhibitors With Efficacy In Vivo for Treatment of Vancomycin‐Resistant Enterococci Septicemia.” Journal of Medicinal Chemistry 68, no. 17: 18597–18624. 40878880 10.1021/acs.jmedchem.5c01584 PMC12527224

[mbo370269-bib-0035] Howerton, A. , M. Patra , and E. Abel‐Santos . 2013. “A New Strategy for the Prevention of *Clostridium difficile* Infection.” Journal of Infectious Diseases 207, no. 10: 1498–1504. 23420906 10.1093/infdis/jit068

[mbo370269-bib-0036] Kotb, A. , N. S. Abutaleb , M. A. Seleem , et al. 2018. “Phenylthiazoles With Tert‐Butyl Side Chain: Metabolically Stable With Anti‐Biofilm Activity.” European Journal of Medicinal Chemistry 151: 110–120. 29605807 10.1016/j.ejmech.2018.03.044 PMC5924651

[mbo370269-bib-0037] Kumar, M. , S. Adhikari , and J. G. Hurdle . 2014. “Action of Nitroheterocyclic Drugs Against *Clostridium difficile* .” International Journal of Antimicrobial Agents 44, no. 4: 314–319. 25129314 10.1016/j.ijantimicag.2014.05.021 PMC4182171

[mbo370269-bib-0038] Lessa, F. C. , L. G. Winston , L. C. McDonald , and T. Emerging Infections Program *C. difficile* Surveillance . 2015a. “Burden of *Clostridium difficile* Infection in the United States.” New England Journal of Medicine 372, no. 24: 2369–2370. 26061850 10.1056/NEJMc1505190 PMC10880113

[mbo370269-bib-0039] Lessa, F. C. , L. G. Winston , and L. C. McDonald , T.E I P C. difficile S. 2015b. “Burden of *Clostridium difficile* Infection in the United States.” New England Journal of Medicine 372, no. 24: 2369–2370. 26061850 10.1056/NEJMc1505190 PMC10880113

[mbo370269-bib-0040] Lev, V. , et al. 2023. “Health Care‐Associated *Clostridioides difficile* Infection: Learning the Perspectives of Health Care Workers to Build Successful Strategies.” American Journal of Infection Control 52, no. 3: 284–292. https://www.ajicjournal.org/article/S0196-6553(23)00552-7/fulltext. 37579972 10.1016/j.ajic.2023.08.008

[mbo370269-bib-0041] Liu, C. , and T. E. Hermann . 1978. “Characterization of Ionomycin as a Calcium Ionophore.” Journal of Biological Chemistry 253, no. 17: 5892–5894. 28319

[mbo370269-bib-0042] Liu, W. C. , D. S. Slusarchyk , G. Astle , W. H. Trejo , W. E. Brown , and E. Meyers . 1978. “Ionomycin, a New Polyether Antibiotic.” Journal of Antibiotics 31, no. 9: 815–819. 711623 10.7164/antibiotics.31.815

[mbo370269-bib-0043] Louie, T. J. , M. A. Miller , K. M. Mullane , et al. 2011. “Fidaxomicin Versus Vancomycin for *Clostridium difficile* Infection.” New England Journal of Medicine 364, no. 5: 422–431. 21288078 10.1056/NEJMoa0910812

[mbo370269-bib-0044] Marshall, J. , K. Y. Wong , C. N. Rupasinghe , et al. 2015. “Inhibition of N‐Methyl‐D‐Aspartate‐Induced Retinal Neuronal Death by Polyarginine Peptides Is Linked to the Attenuation of Stress‐Induced Hyperpolarization of the Inner Mitochondrial Membrane Potential.” Journal of Biological Chemistry 290, no. 36: 22030–22048. 26100636 10.1074/jbc.M115.662791 PMC4571956

[mbo370269-bib-0045] McDonald, L. C. , D. N. Gerding , S. Johnson , et al. 2018. “Clinical Practice Guidelines for Clostridium Difficile Infection in Adults and Children: 2017 Update by the Infectious Diseases Society of America (IDSA) and Society for Healthcare Epidemiology of America (SHEA).” Clinical Infectious Diseases 66, no. 7: e1–e48. 29462280 10.1093/cid/cix1085 PMC6018983

[mbo370269-bib-0046] Morgan, A. J. , and R. Jacob . 1994. “Ionomycin Enhances Ca^2+^ Influx by Stimulating Store‐Regulated Cation Entry and Not by a Direct Action at the Plasma Membrane.” Biochemical Journal 300, no. 3: 665–672. 8010948 10.1042/bj3000665 PMC1138219

[mbo370269-bib-0047] Naclerio, G. A. , N. S. Abutaleb , D. Li , M. N. Seleem , and H. O. Sintim . 2020. “Ultrapotent Inhibitor of *Clostridioides difficile* Growth, Which Suppresses Recurrence In Vivo.” Journal of Medicinal Chemistry 63, no. 20: 11934–11944. 32960605 10.1021/acs.jmedchem.0c01198 PMC9064041

[mbo370269-bib-0048] Nosengo, N. 2016. “Can You Teach Old Drugs New Tricks?” Nature 534, no. 7607: 314–316. 27306171 10.1038/534314a

[mbo370269-bib-0049] Pal, R. , A. I. M. Athamneh , R. Deshpande , et al. 2023. “Probiotics: Insights and New Opportunities for *Clostridioides difficile* Intervention.” Critical Reviews in Microbiology 49, no. 3: 414–434. 35574602 10.1080/1040841X.2022.2072705 PMC9743071

[mbo370269-bib-0050] Pal, R. , M. Dai , and M. N. Seleem . 2021. “High‐Throughput Screening Identifies a Novel Natural Product‐Inspired Scaffold Capable of Inhibiting *Clostridioides difficile* In Vitro.” Scientific Reports 11, no. 1: 10913. 34035338 10.1038/s41598-021-90314-3 PMC8149678

[mbo370269-bib-0051] Pal, R. , and M. N. Seleem . 2020. “Screening of Natural Products and Approved Oncology Drug Libraries for Activity Against *Clostridioides difficile* .” Scientific Reports 10, no. 1: 5966. 32249833 10.1038/s41598-020-63029-0 PMC7136261

[mbo370269-bib-0052] Pal, R. , and M. N. Seleem . 2022. “Discovery of a Novel Natural Product Inhibitor of Clostridioides Difficile With Potent Activity In Vitro and In Vivo.” PLoS One 17, no. 8: e0267859. 35939437 10.1371/journal.pone.0267859 PMC9359557

[mbo370269-bib-0053] Pal, R. , and M. N. Seleem . 2023. “Antisense Inhibition of RNA Polymerase α Subunit of *Clostridioides difficile* .” Microbiology Spectrum 11, no. 5: e0175523. 37772833 10.1128/spectrum.01755-23 PMC10581251

[mbo370269-bib-0054] Qian, Y. , B. T. Birhanu , J. Yang , et al. 2023. “A Potent and Narrow‐Spectrum Antibacterial Against *Clostridioides difficile* Infection.” Journal of Medicinal Chemistry 66, no. 20: 13891–13899. 37732641 10.1021/acs.jmedchem.3c01249 PMC11681498

[mbo370269-bib-0055] Rosch, J. W. , J. Sublett , G. Gao , Y. D. Wang , and E. I. Tuomanen . 2008. “Calcium Efflux Is Essential for Bacterial Survival in the Eukaryotic Host.” Molecular Microbiology 70, no. 2: 435–444. 18761687 10.1111/j.1365-2958.2008.06425.x PMC2577294

[mbo370269-bib-0056] Roshan, N. , T. V. Riley , D. R. Knight , and K. A. Hammer . 2018. “Effect of Natural Products on the Production and Activity of *Clostridium difficile* Toxins In Vitro.” Scientific Reports 8, no. 1: 15735. 30356168 10.1038/s41598-018-33954-2 PMC6200812

[mbo370269-bib-0058] Roshan, N. , T. V. Riley , D. R. Knight , J. H. Steer , and K. A. Hammer . 2019. “Natural Products Show Diverse Mechanisms of Action Against *C. difficile* .” Journal of Applied Microbiology 126, no. 2: 468–479. 10.1111/jam.14152. 30412324

[mbo370269-bib-0059] Rudrapal, M. , S. J. Khairnar , and A. G. Jadhav . 2020. Drug Repurposing (DR): An Emerging Approach in Drug Discovery. In (Ed.), Drug Repurposing ‐ Hypothesis, Molecular Aspects and Therapeutic Applications. IntechOpen.

[mbo370269-bib-0060] Seleem, M. A. , N. S. Abutaleb , H. T. Hussein , et al. 2026. “Synthesis, Antimicrobial Activity, and Preliminary Mechanistic Studies of Phenazine Sulfonamides.” Bioorganic & Medicinal Chemistry 132: 118438. 41092669 10.1016/j.bmc.2025.118438

[mbo370269-bib-0061] Sexton, J. , Repurposing Generic Drugs Can Reduce Time and Cost To Develop New Treatments – But Low Profitability Remains A Barrier. 2022.

[mbo370269-bib-0062] Smith, A. B. , J. Soto Ocana , and J. P. Zackular . 2020. “From Nursery to Nursing Home: Emerging Concepts in *Clostridioides difficile* Pathogenesis.” Infection and Immunity 88, no. 7. 10.1128/iai.00934-19. PMC7309631 32122939

[mbo370269-bib-0063] Sorg, J. A. , and A. L. Sonenshein . 2008. “Bile Salts and Glycine as Cogerminants for *Clostridium difficile* Spores.” Journal of Bacteriology 190, no. 7: 2505–2512. 18245298 10.1128/JB.01765-07 PMC2293200

[mbo370269-bib-0064] Stolz, B. J. , A. Abouelkhair , N. S. Abutaleb , and M. N. Seleem . 2026. “Evaluation of the Antibacterial Activity of the Natural Product α‐Mangostin Against *Clostridioides difficile* .” PLoS One 21, no. 2: e0341857. 41642790 10.1371/journal.pone.0341857 PMC12875497

[mbo370269-bib-0065] Stolz, B. J. , A. A. Abouelkhair , and M. N. Seleem . 2024. “Screening Novel Antiviral Compounds to Treat *Clostridioides difficile* Infections.” PLoS One 19, no. 12: e0309624. 39671442 10.1371/journal.pone.0309624 PMC11642915

[mbo370269-bib-0066] Stratton, C. W. 2003. “Dead Bugs Don't Mutate: Susceptibility Issues in the Emergence of Bacterial Resistance.” Emerging Infectious Diseases 9, no. 1: 10–16. 12533275 10.3201/eid0901.020172 PMC2873758

[mbo370269-bib-0067] Tsigrelis, C. 2020. “Recurrent *Clostridioides difficile* Infection: Recognition, Management, Prevention.” Cleveland Clinic Journal of Medicine 87, no. 6: 347–359. 32487555 10.3949/ccjm.87gr.20001

[mbo370269-bib-0068] Tyrrell, K. L. , D. M. Citron , Y. A. Warren , H. T. Fernandez , C. V. Merriam , and E. J. C. Goldstein . 2006. “In Vitro Activities of Daptomycin, Vancomycin, and Penicillin Against *Clostridium difficile*, *C. perfringens*, Finegoldia Magna, and Propionibacterium Acnes.” Antimicrobial Agents and Chemotherapy 50, no. 8: 2728–2731. 16870765 10.1128/AAC.00357-06 PMC1538685

[mbo370269-bib-0069] Vardakas, K. Z. , K. A. Polyzos , K. Patouni , P. I. Rafailidis , G. Samonis , and M. E. Falagas . 2012. “Treatment Failure and Recurrence of Clostridium Difficile Infection Following Treatment With Vancomycin or Metronidazole: A Systematic Review of the Evidence.” International Journal of Antimicrobial Agents 40, no. 1: 1–8. 22398198 10.1016/j.ijantimicag.2012.01.004

[mbo370269-bib-0070] Vedantam, G. , A. Clark , M. Chu , R. McQuade , M. Mallozzi , and V. K. Viswanathan . 2012. “Clostridium Difficile Infection: Toxins and Non‐Toxin Virulence Factors, and Their Contributions to Disease Establishment and Host Response.” Gut Microbes 3, no. 2: 121–134. 22555464 10.4161/gmic.19399 PMC3370945

[mbo370269-bib-0071] Viswanathan, V. K. , M. Mallozzi , and G. Vedantam . 2010. “ *Clostridium difficile* Infection: An Overview of the Disease and Its Pathogenesis, Epidemiology and Interventions.” Gut Microbes 1, no. 4: 234–242. 21327030 10.4161/gmic.1.4.12706 PMC3023605

[mbo370269-bib-0072] Yadlapati, S. , S. A. Jarrett , K. B. Lo , J. Sweet , and T. A. Judge . 2021. “Examining the Rate of Clostridioides (Formerly Clostridium) Difficile Infection Pre‐ and Post‐COVID‐19 Pandemic: An Institutional Review.” Cureus 13, no. 12: e20397. 35036227 10.7759/cureus.20397 PMC8754356

[mbo370269-bib-0073] Yamaguchi, T. , H. Konishi , K. Aoki , Y. Ishii , K. Chono , and K. Tateda . 2020a. “The Gut Microbiome Diversity of *Clostridioides difficile*‐Inoculated Mice Treated With Vancomycin and Fidaxomicin.” Journal of Infection and Chemotherapy 26, no. 5: 483–491. 32165071 10.1016/j.jiac.2019.12.020

[mbo370269-bib-0074] Yamaguchi, T. , H. Konishi , K. Aoki , Y. Ishii , K. Chono , and K. Tateda . 2020b. “The Gut Microbiome Diversity of *Clostridioides difficile*‐Inoculated Mice Treated With Vancomycin and Fidaxomicin.” Journal of Infection and Chemotherapy 26, no. 5: 483–491. 32165071 10.1016/j.jiac.2019.12.020

[mbo370269-bib-0075] Zhanel, G. G. , A. J. Walkty , and J. A. Karlowsky . 2015. “Fidaxomicin: A Novel Agent for the Treatment of *Clostridium difficile* Infection.” Canadian Journal of Infectious Diseases and Medical Microbiology 26, no. 6: 305–312. 26744587 10.1155/2015/934594 PMC4692299

