# Supplementary material for: Ionomycin Exhibits Potent and Selective Bactericidal Activity Against Clostridioides difficile Through Calcium‐Dependent Membrane Disruption
Source: Microbiologyopen. 2026 Mar 17;15(2):e70269. doi: 10.1002/mbo3.70269 (PMC13140665; doi:10.1002/mbo3.70269)
Supplement: Supplementary file 1 — Figure S1: Evaluation of Ionomycin's Ability to Inhibit C. difficile Spore Germination and control anticlostridial agents against C. difficile ATCC 43255. Vero cells cytotoxicity after challenge with the supernatants of spores incubated with test agents for 2 (A), 8 (B), and 14 days (C). Supernatants were collected, filtered, mixed with DMEM (1:9) and added Vero cell monolayers to determine the cytotoxic impact of toxins produced after germination of C. difficile spores. The cell rounding was analyzed by the confocal microscopy and the cell cytotoxicity (average ± standard deviation) was calculated. The data were analyzed via one‐way ANOVA with post hoc Dunnett's test for multiple comparisons (P< 0.0001). Asterisks indicate statistically significant difference between the data of cell viability for different treatment as compared to the negative control (spores with germinant). Table S1: Description of C. difficile strains used in the study. Table S2: Description of the gut microbiota strains used in the study. [file MBO3-15-e70269-s001.docx]

**Supplemental Material**

**Ionomycin Exhibits Potent and Selective Bactericidal Activity against *Clostridioides difficile* through Calcium-Dependent Membrane Disruption**

Ahmed A. Abouelkhair^1,2^, Nader S. Abutaleb^1,2^, and Mohamed N. Seleem^1,2*^

^1^Department of Biomedical Sciences and Pathobiology, Virginia-Maryland College of Veterinary Medicine, Virginia Polytechnic Institute and State University; Blacksburg, VA, 24061, USA.

^2^Center for One Health Research, Virginia Polytechnic Institute and State University, Blacksburg, VA 24061, USA.

***Corresponding Author:**

Mohamed N. Seleem

Department of Biomedical Sciences and Pathobiology

Virginia-Maryland College of Veterinary Medicine

Virginia Polytechnic Institute and State University

1410 Prices Fork Rd, Blacksburg, VA, 24061, USA

Phone: 540-231-7173

Email: [naguieb@vt.edu](mailto:naguieb@vt.edu)

**Table S1. Description of *C. difficile* strains used in the study**

| **No.** | ***C. difficile* strains** | **Alternate designation** | **Source** | **Characters** |
| --- | --- | --- | --- | --- |
| 1 | **NR-49279 (Ribotype - 027)** | BEI; Isolate 20100211 | **2010**: stool of a young girl patient in New York, USA, who had a community-associated (CA) *C. difficile* infection. | It is positive for *tcd*A*, tcd*B*, tcd*C*,* and binary toxin (CDT). |
| 2 | **NR-49281 (Ribotype - 027)** | BEI; Isolate 20110052 | **2010**: The stool of an older man patient in northeastern USA who had an infection of *C. difficile* linked to healthcare (HA). | It is positive for *tcd*A*, tcd*B*, tcd*C*,* and binary toxin (CDT). |
| 3 | **NR-49283 (Ribotype - 027)** | BEI; Isolate 20120013 | **2011**: stool of a young man patient in northeastern USA who had a community-associated (CA) *C. difficile* infection. | It is positive for *tcd*A*, tcd*B*, tcd*C*,* and binary toxin (CDT). |
| 4 | **NR-49284 (Ribotype - 027)** | BEI; Isolate 20120015 | **2011**: stool of an older man patient in New York, USA, who had an infection with *C. difficile* that was community-associated (CA). | It is positive for *tcd*A*, tcd*B*, tcd*C*,* and binary toxin (CDT). |
| 5 | **NR-49288 (Ribotype - 027)** | BEI; Isolate 20110870 | **2011**: stool of a female patient in her young adult years who had a *C. difficile* infection linked to healthcare (HA) in Tennessee, USA. | It is positive for *tcd*A*, tcd*B*, tcd*C*,* and binary toxin (CDT). |
| 6 | **NR-49291 (Ribotype - 027)** | BEI; Isolate 20120236 | **2011**: stool of an elderly female patient in the Midwest of the United States who had a community-associated (CA) *C. difficile* infection. | It is positive for *tcd*A*, tcd*B*, tcd*C*,* and binary toxin (CDT). |
| 7 | **NR-49303 (Ribotype - 020)** | BEI; Isolate 20120041 | **2011**: stool of an elderly female patient in New York, USA, who had a community-associated (CA) *C. difficile* infection. | It is positive for *tcd*A*, tcd*B*, tcd*C*,* but negative with the binary toxin (CDT). |
| 8 | **NR-49307 (Ribotype - 002)** | BEI; Isolate 20120020 | **2011**: stool of an older female patient in northeastern USA who had a community-associated (CA) *C. difficile* infection. | It is positive for *tcd*A*, tcd*B*, tcd*C*,* but negative with the binary toxin (CDT). |
| 9 | **NR-49309**  **(Ribotype - 002)** | BEI; Isolate 20120190 | **2011**: stool of an older female patient in Connecticut, USA, who had an infection with *C. difficile* that was community-associated (CA). | It is positive for *tcd*A*, tcd*B*, tcd*C*,* but negative with the binary toxin (CDT). |
| 10 | **NR-49313 (Ribotype - 017)** | BEI; Isolate 20110963 | **2011**: stool of an older female patient in Minnesota, USA, who had a *C. difficile* infection that was healthcare-associated (HA). | It is positive for *tcd*A*, tcd*B*, tcd*C*,* but negative with the binary toxin (CDT). |
| 11 | **NR-49317 (Ribotype - 024)** | BEI; Isolate 20111163 | **2011**: stool from an older female patient in northeastern USA who had a community-associated (CA) *C. difficile* infection. | It is positive for *tcd*A*, tcd*B*, tcd*C*,* and binary toxin (CDT). |
| 12 | **NR-49318 (Ribotype - 106)** | BEI; Isolate 20110973 | **2011**: stool of a young girl patient in the Midwest USA who had a community-associated (CA) *C. difficile* infection. | It is positive for *tcd*A*, tcd*B*, tcd*C*,* but negative with the binary toxin (CDT). |
| 13 | CD-**2** (Ribotype - **056**) | CDC; Isolate 1068 | Isolated from Homosapien (Unknown) in 2016, USA | It is positive for *tcdA, tcdB,* but negative with *cdtA, cdtB* |
| 14 | CD-**3** (Ribotype - **015**) | CDC; Isolate 1069 | Isolated from Homosapien (Unknown) in 2016, USA | It is positive for *tcdA, tcdB,* but negative with *cdtA, cdtB* |
| 15 | CD-**4** (Ribotype - **002**) | CDC; Isolate 1070 | Isolated from Homosapien (Unknown) in 2016, USA | It is positive for *tcdA, tcdB,* but negative with *cdtA, cdtB* |
| 16 | CD-**5** (Ribotype - **027**) | CDC; Isolate 1071 | Isolated from Homosapien (Unknown) in 2016, USA | It is positive for *tcdA, tcdB,* *cdtA,* and *cdtB* |
| 17 | CD-**7** (Ribotype - **020**) | CDC; Isolate 1073 | Isolated from Homosapien (Unknown) in 2016, USA | It is positive for *tcdA, tcdB,* but negative with *cdtA, cdtB* |
| 18 | CD-**8** (Ribotype - **002**) | CDC; Isolate 1074 | Isolated from Homosapien (Unknown) in 2016, USA | It is positive for *tcdA, tcdB,* but negative with *cdtA, cdtB* |
| 19 | CD-**9** (Ribotype - **019**) | CDC; Isolate 1075 | Isolated from Homosapien (Unknown) in 2016, USA | It is positive for *tcdA, tcdB,* *cdtA,* and *cdtB* |
| 20 | CD-**16** (Ribotype - **054**) | CDC; Isolate 1082 | Isolated from Homosapien (Unknown) in 2016, USA | It is positive for *tcdA, tcdB,* but negative with *cdtA, cdtB* |
| 21 | CD-**17** (Ribotype - **078**) | CDC; Isolate 1083 | Isolated from Homosapien (Unknown) in 2016, USA | It is positive for *tcdA, tcdB,* *cdtA,* and *cdtB* |
| 22 | CD-**18** (Ribotype - **002**) | CDC; Isolate 1084 | Isolated from Homosapien (Unknown) in 2016, USA | It is positive for *tcdA, tcdB,* but negative with *cdtA, cdtB* |
| 23 | CD-**19** (Ribotype - 106) | CDC; Isolate 1085 | Isolated from Homosapien (Unknown) in 2016, USA | It is positive for *tcdA, tcdB,* but negative with *cdtA, cdtB* |
| 24 | CD-2**0** (Ribotype - **015**) | CDC; Isolate 1086 | Isolated from Homosapien (Unknown) in 2016, USA | It is positive for *tcdA, tcdB,* but negative with *cdtA, cdtB* |
| 25 | CD-**25** (Ribotype - **014**) | CDC; Isolate 1091 | Isolated from Homosapien (Unknown) in 2016, USA | It is positive for *tcdA, tcdB,* but negative with *cdtA, cdtB* |
| 26 | CD-27 (Ribotype - 106) | CDC; Isolate 1093 | Isolated from Homosapien (Unknown) in 2016, USA | It is positive for *tcdA, tcdB,* but negative with *cdtA, cdtB* |
| 27 | **ATCC 630 (Ribotype - 012)** | ATCC; Isolate BAA-1382 | Switzerland | It is positive for *tcdA, tcdB,* but negative with *cdtB* |
| 28 | **ATCC 43255 (Ribotype - 087)** | ATCC; Isolate VPI 10463 | Abdominal wound (Ribotype-087) | It is positive for *tcdA, tcdB,* but negative with *cdtB* |
| 29 | **ATCC BAA-1870 (Ribotype - 027)** | ATCC; Isolate 4118 | Clinical isolate (Ribotype-027) | It is positive for *tcdA, tcdB,* and *cdtB* |
| 30 | **ATCC 9689 (Ribotype - 001)** | ATCC | Clinical isolate (Ribotype-001) | It is positive for *tcdA, tcdB,* but negative with *cdtB* |

**CDC**; The Centers for Disease Control and Prevention.

**BEI Resources**; The Biodefense and Emerging Infections Research Resources Repository.

**ATCC**; The American Type Culture Collection.

**Table S2. Description of the gut microbiota strains used in the study.**

| **No** | **Microbiota strains** | **Alternate designation** | **Source** |
| --- | --- | --- | --- |
| 1 | *Bifidobacterium breve* HM-856 | HPH0326 | A biopsy of ileo-anal pouch mucosa of a human, USA |
| 2 | *Bacteroides finegoldii* HM-727 | CL09T03C10 | Healthy adult human feces in Massachusetts, USA. |
| 3 | *Bacteroides stercoris* HM-1036 | CC31F | **2010**: from colonic biopsy tissue of a human subject in Victoria, Canada. |
| 4 | *Bacteroides fragilis* HM-20 | 3_1_12 | 2007: from the transverse colon of a healthy 52-year-old female undergoing a colon cancer screen procedure, Canada. |
| 5 | *Bacteroides fragilis* HM-710 | CL07T12C05 | Healthy adult human feces in Massachusetts, USA. |
| 6 | *Bacteroides fragilis* HM-714 | CL03T12C07 | Healthy adult human feces in Massachusetts, USA. |
| 7 | *Bacteroides fragilis* HM- 711 | CL05T00C42 | Healthy adult human feces in Massachusetts, USA. |
| 8 | *Bacteroides caccae* HM-728 | CL03T12C61 | Healthy adult human feces in Massachusetts, USA. |
| 9 | *Bacteroides dorei* HM-29 | 5_1_36/D4 | **2007**: from inflamed biopsy tissue taken from the terminal ileum of a 45-year-old male patient with Crohn’s disease, Canada. |
| 10 | *Lactobacillus gasseri* ATCC 19992 | 1SL4 [F 164, VPI 6033] | Feces |
| 11 | *Lacticaseibacillus rhamnosus* HM-106 | LMS2-1 | LMS2-1 is a human gastrointestinal isolate. |

**
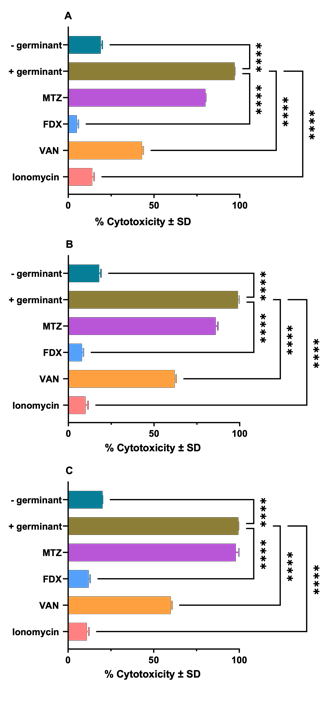
**

| Ionomycin | VAN | FDX | MTZ | + germinant | -germinant | Untreated |
| --- | --- | --- | --- | --- | --- | --- |
| 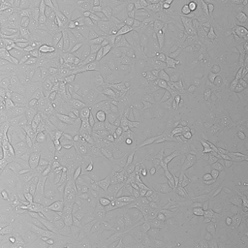 | **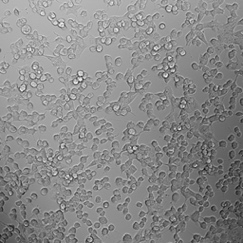** | **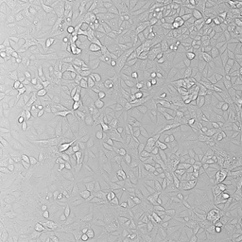** | **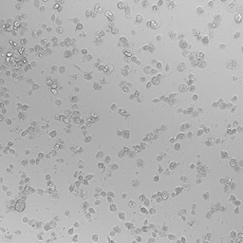** | **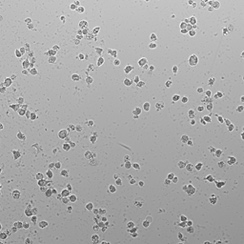** | **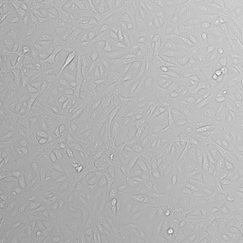** | **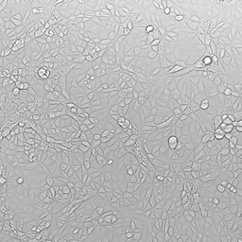** |
| 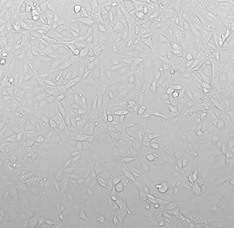 | **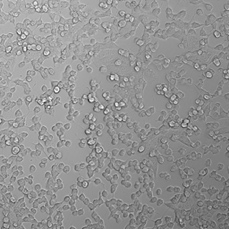** | **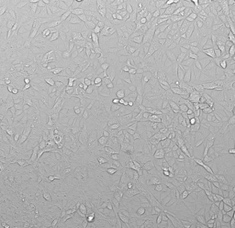** | **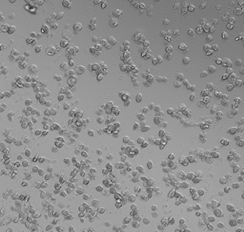** | **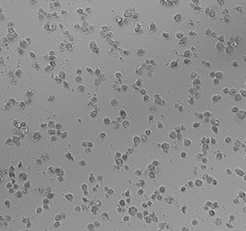** | **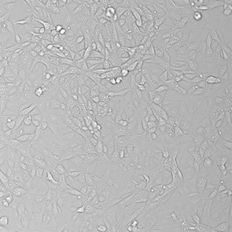** | **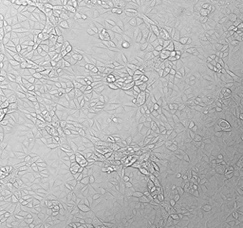** |
| 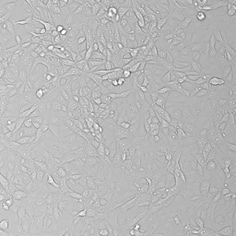 | **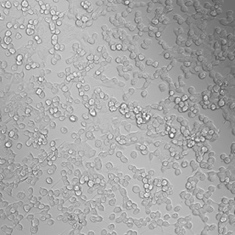** | **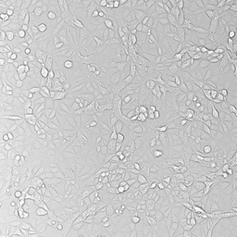** | 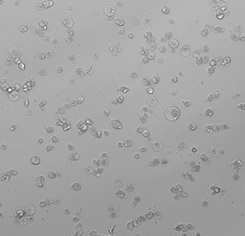 | **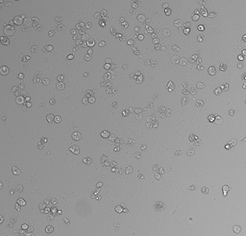** | **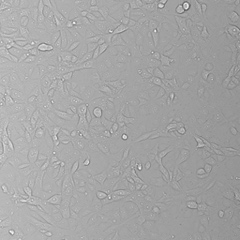** | **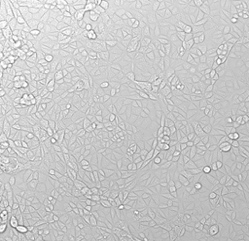** |

**Figure S1.** **Evaluation of Ionomycin’s Ability to Inhibit *C. difficile* Spore Outgrowth and control anticlostridial agents against *C. difficile* ATCC 43255.** Vero cells cytotoxicity after challenge with the supernatants of spores incubated with test agents for 2- (**A**), 8- (**B**), and 14-days (**C**). Supernatants were collected, filtered, mixed with DMEM (1:9) and added Vero cell monolayers to determine the cytotoxic impact of toxins produced after germination of *C. difficile* spores. The cell rounding was analyzed by the the differential interference contrast (DIC) microscope and the cell cytotoxicity (average ± standard deviation) was calculated. The data were analyzed via one-way ANOVA with post hoc Dunnett’s test for multiple comparisons (*P< 0.0001*). Asterisks indicate statistically significant difference between the data of cell viability for different treatment as compared to the negative control (spores with germinant).
